# Mobile Emergency Care Service: direct costs for personnel, materials, medications and solutions*

**DOI:** 10.1590/1980-220X-REEUSP-2024-0156en

**Published:** 2025-08-11

**Authors:** Luciana Mota Leonor do Nascimento, Antônio Fernandes Costa Lima

**Affiliations:** 1 Universidade de São Paulo, Escola de Enfermagem, Programa de Pós-Graduação em Gerenciamento em Enfermagem, São Paulo, SP, Brazil.; 2 Universidade de São Paulo, Escola de Enfermagem, Departamento de Orientação Profissional, São Paulo, SP, Brazil.

**Keywords:** Pre-Hospital Care, Emergency Medical Services, Emergency Nursing, Ambulances, Direct Service Costs

## Abstract

**Objective:**

To identify average direct costs of personnel, materials, medications and solutions required for pre-hospital care by the Mobile Emergency Care Service in a city in São Paulo.

**Method:**

A bottom-up microcosting by retrospective absorption in the form of a single case study. Data were collected from a sample of 380 Systematized Care Records for 2022. Costs were obtained by multiplying the time spent per professional by direct labor unit cost, adding input costs.

**Results:**

In Advanced Life Support teams, average direct costs for doctors and nurses were US$10.98, and for materials, medications and solutions, US$8.72, totaling US$19.70/service. In Basic Life Support teams, average direct costs for nursing technicians were US$1.11, and for materials, medications and solutions, US$2.10, totaling US$3.21/service.

**Conclusion:**

Mobile pre-hospital care costs can support strategic care and management decision-making, aiming at maintaining its financial and economic sustainability.

## INTRODUCTION

In Brazil, mobile pre-hospital emergency services are considered essential, as they enable early recognition of an injury and the provision of necessary interventions to save lives, with the sending of adequate resources, immediate care and transportation to a reference location integrated into the Brazilian Health System (In Portuguese, *Sistema* Único *de Saúde* – SUS)^(1)^.

Mobile pre-hospital care (PHC) for emergency care has been increasingly requested, due to the growing number of accidents, increase in violence and inefficiency in structuring the healthcare services network^(2)^. In this context, the Mobile Emergency Care Service (In Portuguese, *Serviço de Atendimento Móvel de Urgência* – SAMU) offers early care to victims of health problems of any nature, reducing the number of deaths and promoting faster recovery by reducing after-effects^(3)^.

Emergency care at SAMU begins at the Emergency Coordination Center (ECC), where calls from the public are received, qualified listening is provided and patients’ risk is assessed. An ECC activates the appropriate mobile unit, either for on-site care, through a Basic Life Support (BLS) or Advanced Life Support (ALS) team, or for referral to the most appropriate healthcare service for the case. In on-site care, the team assesses victims and performs first aid, following the guidelines of coordinating physicians^(3)^.

At SAMU, the ALS team is made up of ambulance driver, general practitioner and nurse, and the BLS team is made up of ambulance driver, nurse or nursing technician or assistant^(4)^. Mobile PHC is based on protocols developed by the Ministry of Health, together with the *Hospital Alemão Oswaldo Cruz*, through the SUS Institutional Development Support Program, which establishes strategic partnerships to promote research, innovation, human resources qualification and health management^(5)^.

Ordinance 2048/MO of 05/11/2002 determines the duties and competencies of healthcare professionals working in SAMU teams, establishing the following composition: a physician technically responsible, qualified to exercise PHC, manager of medical activities; coordinating physicians, responsible for managing care operationalization; interventionist physicians, responsible for patient care, from resuscitation to stabilization, at the scene of the incident and during transport; nurse, responsible for managing nursing care activities; interventionist nurses, responsible for nursing care necessary for patient resuscitation and stabilization, at the scene of the incident until transport; and nursing technicians, responsible for technical level care, under the supervision of a nurse^(2)^.

The services provided by SAMU teams can be classified as primary, when the request is made by a user, and secondary, when the request is made by a healthcare service so that patients can be transferred to another health unit of greater complexity, aiming at continuity of treatment. Regardless of the type of care (ALS or BLS), teams’ responsibility is highlighted when providing assistance to individuals suffering from health problems, stabilizing and proceeding with the safe and quality referral to health facilities^(6)^.

The data generated in each service (information about the patient, the occurrence and the procedures performed) are recorded in Systematized Service Records (SSRs), which provide legal support to the team and the patient, as well as visibility to the service provided, supporting epidemiological analyses essential to the improvement of the local PHC and its continuity^(7)^.

In 2023, SAMU was present in 67.3% of Brazilian municipalities, serving 85% of the population^(8)^. Its funding has been a tripartite responsibility between the union, with 50% of expenses, states and municipalities, which cover expenses according to the agreement stipulated in each Bipartite Intermanagerial Commission^(3)^.

For the construction of Emergency Medical Coordination Centers (EMCCs) and expansion of existing ones, resources comply with the following parameters: R$216,000.00, when the municipal population is up to 350,000 inhabitants; R$350,000.00, when the municipal population is from 350,001 to 3,000,000 inhabitants; R$440,000.00, when the municipal population is above 3,000,000 inhabitants^(9)^. For monthly costs, the following parameters apply: R$54,600.00 per month, when qualified; and R$68,386.50 per month, when qualified and with up to 350,000 inhabitants^(10)^.

The values for the acquisition of technology and IT equipment from EMCCs cover up to R$96,847.21, when the municipal population is up to 350,000 inhabitants; up to R$288,817.15, when the municipal population is between 350,001 and 10,000,000 inhabitants; and R$302,902.15, when the municipal population is above 10,000,001 inhabitants^(9)^.

For the maintenance of effectively implemented teams, the values of R$17,062.50 per month per qualified BLS unit were established; R$28,494.70 per month per qualified and qualified BLS unit; R$50,050.00 per month per qualified ALS unit; R$62,687.30 per month per qualified and qualified ALS unit; R$54,600.00 per month (population of up to 350,000 inhabitants) per qualified EMCC unit; and R$68,386.50 per month (population of up to 350,000 inhabitants) per qualified EMCC unit^(9)^.

The monthly cost allocated to SAMU is directly linked to the monthly records of services, and may be suspended when qualification and accreditation requirements are not met, the number of services is lower than that established by the specific ordinance of the Department of Healthcare, due to the absence of records of services for three consecutive months, or irregularities are found in the service^(9)^.

Municipal management is responsible for allocating the material, human, and financial resources necessary for SAMU’s viability so that the quality of care provided is not compromised^(3)^. It is worth noting that municipalities were encouraged to implement SAMU due to the financial incentive for its implementation, in addition to the distribution of equipped vehicles^(9)^. A study carried out in 2022 showed that SAMU registered 133.4 million procedures between 2015 and 2019. The population covered increased by 9.7%; the number of calls via 192 increased by 26.5%; and the total number of procedures performed by BLS and ALS increased by 28.5%^(11)^.

The national policy for expanding emergency care services requires funding that is compatible with the SUS requirements, referring to the sufficiency and adequate distribution of resources^(12)^. It is known that good management is a strategic basis for regulating and organizing the care provided by SAMU. However, there are some problems in the health system that can affect quality of care, such as inadequate investment, precarious resources, lack of infrastructure, lack of human resources and lack of vehicle maintenance, making it necessary for managing authorities to commit to making the required investments^(13)^.

A study carried out in 2022, in the Greater ABC region, in São Paulo, showed that managers highlighted the increase in demands for SAMU, but without an increase in financial support. They also highlighted the lack of equipment and supplies necessary for care, reinforcing that the lack of resources could compromise the achievement of favorable results^(14)^.

Given that financial resources are limited and that demands for care are increasing, an important step in cost management is to determine costs associated with PHC. Thus, the objective was to identify the average direct costs (ADCs) of personnel, materials, medications and solutions required by PHC of SAMU in a city in São Paulo.

## METHOD

### Study Design

This is retrospective bottom-up absorption microcosting in the form of a single case study*.*

### Place

The research was conducted at the SAMU Regional Base of a Brazilian municipality in the state of São Paulo. In 2022, the aforementioned SAMU received 85,709 calls (75,429 clinical, 5,964 trauma, 1,202 obstetric, 1,137 pediatric and 1,977 psychiatric), which resulted in 28,267 on-site care, 26,177 of which were BLS and 2,090 of ALS.

### Sample and Selection Criteria

From the population of SSRs (N = 28,267), for the year 2022, a statistician calculated the minimum sample size to be analyzed, using the sample sizing methodology to estimate an average. Since there was no cost standard deviation (SD) estimate, the margin of error ε = 0.1σ was assumed, i.e., a maximum of 10% of the SD. Thus, the sample size calculation of 380 SSRs was obtained, 188 related to services provided by ALS teams and 192 by BLS teams.

The SSRs that were adequately filled out with the variables identifying the call (city, date, time, vehicle called) and patient (sex and age), location of occurrence, time spent and supplies (materials, medications and solutions) consumed were selected. SSRs that were incompletely filled out were excluded.

### Data Collection

All data, including those related to the time spent on care provided by healthcare professionals, were collected from May to August 2023 through electronic access to the sample of SSRs completed by BLS and ALS teams in the Municipal Health Department Administrative Coordination room.

The study consisted of bottom-up absorption microcosting, in which the actual resources consumed by a patient or healthcare service were consulted, determining the real cost of offering healthcare by a provider. The bottom-up microcosting method was chosen because it allows for the definition, in detail, of all the variables that make up costs, based on individual data on the treatment provided to patients, with the review of medical records or clinical records specific to the study^(15)^.

Absorption costing corresponds to the allocation of production costs to the goods produced. In this way, all expenses related to production are shared between the services or products performed; through it, manufacturing costs are assigned to product cost^(16)^.

Thus, direct costs were calculated, defined as those that can be identified and quantified, which refer to a monetary value relative to the consumption of direct labor (DL), materials, medications and solutions^(16)^. DL refers to professionals who work on a given product/service, as long as it is possible to measure the time spent on its completion. It consists of wage, charges, vacations and 13^th^ wage^(16)^.

Healthcare professionals’ DL was calculated based on wage information provided by the municipal Human Resources Department (HRD) responsible for the SAMU study field. For costing purposes, the values, obtained in *reais* (R$), were converted to US dollars (US$) at an average rate of US$4.75/R$1.00, based on quotations from January to December 2022 provided by the *Banco Central do Brasil*.

Thus, the weighted average of physicians’ payroll corresponded to US$252.63/12 hours, US$21.05/hour and US$0.35/minute; nurses’ payroll corresponded to US$105.26/12 hours, US$8.77/hour and US$0.15/minute; and nursing technicians’ payroll corresponded to US$52.63/12 hours, US$4.38/hour and US$0.07/minute. The information regarding the costs of the latest acquisitions of material inputs, medications and solutions was made available by the Purchasing/Warehouse Department.

It should be noted that, during the period in which the study was conducted, driving the ALS and BLS vehicles was an activity delegated to military firefighters paid by the State, whose wage information was not available to the aforementioned HRD. It was not possible to calculate vehicles’ fuel consumption, given that all vehicles in the municipality were fueled at the same location and payment was made in a single manner, making it impossible to distinguish vehicles from other vehicles.

To obtain ADC of services recorded by ALS and BLS teams, the equation was used^(17)^ $\bar{C\left(P_{\text{τ}}\right)}=\sum_{k=1}^{n}\left(\bar{q_{k}.}\;\bar{Pu_{k}}\right)+\sum_{k=1}^{n}\left(\bar{qs_{k}.}\;\bar{Psu_{k}}\right)+\sum_{c=1}^{n}\left(\bar{t_{c}.}\;\bar{su_{c}}\right)$ , covering the following variables: $\bar{qm_{k}}$ (average amount of materials); $\bar{Pmu_{k}}$ (unit price of each material); $\bar{qs_{k}}$ (average amount of solutions/medications); $\bar{Psu_{k}}$ (unit price of each solution/medication); $\bar{t_{c}}$ (average time spent by each professional); $\bar{Su_{c}}$ (unit wage mass of each workforce)^(17)^. In summary, the time spent by the performing professional was multiplied, according to the category, by the cost of the respective DL, plus the cost of materials, medications and solutions^(17)^ consumed in each ALS and BLS service.

### Data Analysis and Treatment

The data were organized in an electronic spreadsheet, using independent double typing, and were treated using descriptive and inferential statistics.

### Ethical Aspects

The Research Ethics Committee of the proposing institution approved, after the consent of the Municipal Health Department responsible for the SAMU studied, this study (Opinion 6,031,451 of April 28, 2023).

## RESULTS

From the sample of 192 SSRs related to the BLS team’s services, the average nursing technician service time was 15.85 minutes. Table 1 shows that the total ADC corresponded to US$3.21/service (SD = 2.61), with greater representation of ADC with materials, medications and solutions (US$2.10–SD = 2.26).

**Table 1:** Distribution of direct labor costs in dollars (US$*) of nursing technicians working in the 192 Basic Life Support team visits according to mean, standard deviation, minimum, maximum, 1^st^ quartile, median and 3^rd^ quartile – São Paulo, SP, Brazil, 2023.

Variables	ADC (US$*)	Standard deviation	Percentage of composition of total ADC (%)	Minimum (US$*)	Maximum (US$*)	1^st^ quartile (US$)	Median (US$)	3^rd^ quartile (US$)
**Total ADC with nursing technician DL**	**1.11**	**0.75**	**34.55%**	0.15	4.82	0.58	0.95	1.41
**Total ADC with materials, medications and solutions**	**2.10**	**2.26**	**65.45%**	0.00	18.18	0.41	1.68	3.05
**Total ADC for BLS service**	**3.21**	**2.61**	**100.00%**	0.15	19.94	0.34	2.39	4.40

Note: *Average conversion rate: R$4.75/US$1.00. *Banco Central*, based on the exchange rate from January to December 2022; DL – direct labor; BLS – Basic Life Support; ADC – average direct cost.

According to the records of materials, medications, and solutions in the 192 SSRs analyzed, the total cost of BLS services was US$396.67 (100.00%), with an ADC of US$2.10 per service. Table 2 shows that, among the 39 items, those that contributed most to the composition of this total cost were cervical collar S/33 services (unit cost: US$3.00; 24.94%), saline solution 0.9% 250 ml/40 services (unit cost: US$0.93; 9.19%), splint M/14 services (unit cost: US$2.57; 9.07%), and cervical collar M/10 services (unit cost: US$3.19; 8.04%).

**Table 2:** Distribution of costs in dollars (US$*) of materials, medications and solutions consumed in the 192 Basic Life Support services according to total cost, percentage of total cost composition, number of services, average direct cost per service and unit value – São Paulo, SP, Brazil, 2023.

Materials, medications and solutions (supplies)	Total cost (US$)	Percentage of composition of total cost (%)	Number of services provided	Input ADC per service (US$)	Input unit cost (US$)
Cervical collar P	98.93	24.94	33	3.00	3.00
0.9% saline solution 250 ml	36.45	9.19	40	0.93	0.93
Wire splint M	35.99	9.07	14	2.57	2.57
Cervical collar M	31.89	8.04	10	3.19	3.19
Macrodrops equipment	22.05	5.56	38	0.61	0.61
High concentration mask	20.77	5.24	8	2.60	2.60
Wire splint – G	18.91	4.77	5	3.78	3.78
0.9% saline solution 500 ml	16.45	4.15	17	0.97	1.10
Wire splint – P	15.24	3.84	6	2.54	2.18
12 cm bandage	13.60	3.43	53	0.26	0.16
Capillary blood glucose test strip	13.41	3.38	93	0.15	0.15
Cotton gauze	11.76	2.96	12	0.98	0.73
Wire splint – GG	8.11	2.05	1	8.11	4.06
Non-needle catheter 20	7.12	1.80	18	0.42	0.42
Non-needle catheter 22	6.25	1.58	15	0.45	0.45
Glass-type nasal catheter	5.46	1.38	32	0.17	0.17
Procedure glove – P (latex/powdered)	4.71	1.19	89	0.05	0.03
50% glucose ampoule 10 ml	4.28	1.08	12	0.36	0.12
Sterile gauze (package)	3.82	0.96	27	0.14	0.14
Procedure glove – M (latex/powdered)	3.69	0.93	74	0.03	0.03
Capillary blood glucose lancet	3.07	0.77	93	0.03	0.03
Triangular bandage – M	2.88	0.73	4	0.72	0.72
Needle catheter 21	2.15	0.54	5	0.43	0.43
Needle catheter 23	2.09	0.53	5	0.42	0.42
Non-needle catheter 18	1.89	0.48	4	0.47	0.47
Procedure glove – G (latex/powdered)	1.30	0.33	28	0.05	0.02
Syringe 20 ml	1.15	0.29	14	0.07	0.07
Needleless catheter 14	0.95	0.24	1	0.95	0.95
Needleless catheter 24	0.49	0.12	1	0.49	0.49
Guedel cannula 4	0.44	0.11	1	0.44	0.44
Dipyrone sodium 500 mg ampoule	0.23	0.06	1	0.23	0.23
Needle 40 × 12	0.20	0.05	15	0.01	0.01
N95 mask	0.20	0.05	1	0.20	0.20
Electrodes for monitoring	0.20	0.05	1	0.20	0.04
Scopolamine butylbromide ampoule	0.20	0.05	1	0.20	0.20
Syringe 10 ml	0.12	0.03	2	0.06	0.04
Captopril 25 mg tablet	0.12	0.03	4	0.02	0.02
Distilled water ampoule 10 ml	0.11	0.03	1	0.11	0.05
Disposable coverall	0.01	0.00	1	0.01	0.01

Note: *Average conversion rate: R$4.75/US$1.00. *Banco Central*, based on the exchange rate from January to December 2022; DL – direct labor; ADC – average direct cost.

Considering the sample of 188 SSRs related to the services provided by ALS teams, Table 3 shows that the total ADC corresponded to US$19.70/service (SD = 36.34), with greater representation of the total ADC with DL from healthcare professionals (US$10.99–SD = 5.81), whose average joint service time corresponded to 22 minutes.

**Table 3:** Distribution of costs in dollars (US$*) of direct labor of healthcare professionals working in the 188 Advanced Life Support services and of supplies consumed according to mean, standard deviation, minimum, maximum, 1^st^ quartile, median and 3^rd^ quartile – São Paulo, SP, Brazil, 2023.

Variables	ADC (US$*)	Standard deviation	Percentage of composition of total ADC (%)	Minimum (US$*)	Maximum (US$*)	1^st^ quartile (US$)	Median (US$)	3^rd^ quartile (US$)
ADC with nurse DL	3.23	2.12	16.40	0.58	15.79	1.90	3.80	3.80
ADC with physician DL	7.75	5.10	39.36	1.40	37.89	4.56	6.67	9.12
**Total ADC with DL**	**10.99**	**5.81**	**55.76**	1.99	53.68	6.46	9.44	12.92
**Total ADC with materials, medications and solutions**	**8.72**	**32.06**	**44.24**	0.27	212.46	2.24	3.02	5.12
**Total ADC for ALS care**	**19.70**	**36.64**	**100.00**	**2.79**	**256.51**	**2.24**	**13.27**	**18.05**

Note: *Average conversion rate: R$4.75/US$1.00. *Banco Central*, based on the exchange rate from January to December 2022; DL – direct labor; ALS – Advanced Life Support; ADC – average direct cost.

According to records of materials, medications and solutions contained in the 188 SSRs, the total cost corresponded to US$1,647.49 (100.00%), with an ADC of US$8.72 per service. According to Table 4, among the 73 items consumed, those that contributed most to the composition of this total cost were intraosseous puncture kit/five consultations (unit cost: US$193.68; 58.78%), 0.9% saline solution - 500 ml/75 consultations (unit cost: US$1.10; 5.33%), cervical collar P/27 consultations (unit cost: US$3.00; 4.91%), macrodrop equipment/130 consultations (unit cost: US$0.61; 4.91%) and high concentration mask/21 consultations (unit cost: US$2.60; 3.31%).

**Table 4:** Distribution of costs in dollars (US$) of materials, medications and solutions consumed in the 188 Advanced Life Support services according to total cost, percentage of total cost composition, number of services, average direct cost per service and unit value – São Paulo, SP, Brazil, 2023.

Materials, medications and solutions (supplies)	Total cost (US$)	Percentage of composition of total cost (%)	Number of services provided	ADC of input per service (US$)	Input unit cost (US$)
Intraosseous puncture kit	968.42	58.78	5	193.68	193.68
Saline solution 0.9% 500 ml	87.75	5.33	75	1.17	1.10
Cervical collar S	80.94	4.91	27	3.00	3.00
Macrodrops equipment	80.87	4.91	130	0.61	0.61
High concentration mask	54.51	3.31	21	2.60	2.60
Ringer’s lactate solution 500 ml	38.50	2.34	22	1.75	1.60
Saline solution 0.9% 250 ml	35.52	2.16	35	1.01	0.93
Wire splint – M	23.13	1.40	8	2.89	2.57
Electrodes for monitoring	20.60	1.25	103	0.20	0.04
Non-needle catheter 20	20.11	1.22	48	0.42	0.42
Cotton gauze	19.10	1.16	16	1.19	0.73
Wire splint – G	18.91	1.15%	4	4.73	3.78
Non-needle catheter 18	15.09	0.92	32	0.47	0.47
Non-needle catheter 22	13.39	0.81	30	0.45	0.45
Glucose solution 5% 250 ml	10.93	0.66	9	1.21	1.09
Bandage 12 cm	9.12	0.55	32	0.29	0.16
Non-needle catheter 16	8.70	0.53	13	0.67	0.67
Capillary blood glucose tape	8.55	0.52	58	0.15	0.15
Procedure glove M (latex/powdered)	8.24	0.50	108	0.08	0.03
Epinephrine 1 mg vial	7.52	0.46	9	0.84	0.21
Procedure glove – P (latex/powdered)	6.95	0.42	89	0.08	0.03
Wire splint P	6.53	0.40	3	2.18	2.18
Orotracheal tube 7.5	6.46	0.39	9	0.72	0.72
Glucose 50% ampoule 10 ml	6.25	0.38	14	0.45	0.12
Suxamethonium chloride 100 mg ampoule	5.91	0.36	2	2.95	2.95
Midazolam 15 mg ampoule	4.21	0.26	7	0.60	0.60
Midazolam 5 mg ampoule	4.04	0.25	5	0.81	0.40
Procedure glove G (latex/powdered)	3.98	0.24	64	0.06	0.02
Dipyrone sodium 500 mg ampoule	3.83	0.23	17	0.23	0.23
Etomidate ampoule 10 ml	3.83	0.23	2	1.91	1.91
Glass-type nasal catheter	3.75	0.23	22	0.17	0.17
No-needle catheter 24	3.45	0.21	7	0.49	0.49
Ondansetron ampoule 2 ml	3.27	0.20	8	0.41	0.41
Tramadol hydrochloride ampoule	3.23	0.20	7	0.46	0.46
Cervical collar M	3.19	0.19	1	3.19	3.19
Haloperidol 5 mg ampoule	3.09	0.19	8	0.39	0.34
Ketoprofen vial ampoule	2.77	0.17	4	0.69	0.69
Sterile gauze (package)	2.68	0.16	14	0.10	0.10
Needle catheter 23	2.51	0.15	6	0.42	0.42
Flumazenil 0.5 mg ampoule	2.37	0.14	2	1.19	1.19
Omeprazole 40 mg ampoule	2.27	0.14	2	1.14	1.14
Triangular bandage – M	2.16	0.13	3	0.72	0.72
Needle catheter 21	2.15	0.13	5	0.43	0.43
Diazepam ampoule	2.15	0.13	10	0.21	0.21
Orotracheal tube 8.0	2.15	0.12	3	0.67	0.67
Lancet for capillary blood glucose	1.95	0.12	58	0.03	0.03
Non-needle catheter 14	1.89	0.12	2	0.95	0.95
Morphine ampoule	1.83	0.11	2	0.92	0.92
Fentanyl citrate 50 mcg ampoule	1.81	0.11	4	0.45	0.45
Clopidogrel 75 mg tablet	1.77	0.11	6	0.29	0.07
Phenytoin ampoule	1.52	0.09	3	1.03	0.60
Amiodarone 50 mg ampoule	1.41	0.09	2	0.71	0.47
Syringe 5 ml	1.37	0.08	33	0.04	0.03
Guedel cannula 4	1.33	0.08	3	0.44	0.44
Syringe 10 ml	1.28	0.08	30	0.04	0.04
Syringe 20 ml	1.28	0.08	15	0.09	0.07
Promethazine hydrochloride ampoule	1.14	0.07	3	0.38	0.38
Needle 40 × 12	0.92	0.06	69	0.01	0.01
Distilled water ampoule 10 ml	0.89	0.05	13	0.07	0.05
Tranexamic acid ampoule	0.86	0.05	1	0.86	0.86
Multi-way connector	0.77	0.05	3	0.26	0.26
Heating blanket – G	0.49	0.03	1	0.49	0.49
Furosemide ampoule	0.47	0.03	1	0.47	0.24
Scopolamine butylbromide ampoule	0.40	0.02	2	0.20	0.20
Needle 25 × 7	0.34	0.02	13	0.03	0.02
N95 mask	0.20	0.01	1	0.20	0.20
Captopril 25 mg tablet	0.19	0.01	6	0.03	0.02
Acetylsalicylic acid 100 mg tablet	0.18	0.01	8	0.02	0.01
Tracheal aspiration tube 10	0.12	0.01	1	0.12	0.12
Diazepam 10 mg tablet	0.07	0.00	4	0.02	0.02
Isosorbide 5 mg tablet	0.04	0.00	3	0.01	0.01
Disposable coverall	0.03	0.00	2	0.01	0.01
Hydrochlorothiazide tablet	0.01	0.00	1	0.01	0.01

Note: *Average conversion rate: R$4.75/US$1.00. Banco Central, based on the exchange rate from January to December 2022; DL – direct labor; ALS – Advanced Life Support; ADC – average direct cost.

The total ADCs for the sample of 380 SSRs corresponded to US$4,319.92, of which US$616.32 were related to 192 BLS services, and US$3,703.60 were related to 188 ALS services. Extrapolating the ADCs to the population of 28,267 SSRs, the total ADCs would correspond, in the year 2022, to US$125,201.17 (100.00%), of which US$84,028.17 (67.11%) were related to 26,177 BLS services, and US$41,173.00 (32.89%) were related to 2,090 ALS services.

## DISCUSSION

In the BLS team’s care, it was found that ADC with materials, medications and solutions was higher than the ADC with nursing technicians’ DL, contributing with 65.45% of the total ADC per care. The most used materials were lancets and capillary blood glucose test strips, consumed in 93 of the 192 care sessions. Recent research, covering different study objects, also indicated the predominance of costs related to material resources^(18,19,20)^.

A single case, quantitative, exploratory-descriptive study, when calculating the ADC of the insertion of long-term central venous catheter in patients undergoing hemodialysis in a public hospital in the state of São Paulo, obtained ADC of US$107.01 (SD = 0.23) with material, US$22.10 (SD = 3.63) with professionals’ DL, US$4.65 (SD = 0.00) with medications and US$0.80 (SD = 0.15) with solutions^(18)^.

Another quantitative, exploratory-descriptive, single-case study that calculated the ADC for prostate cancer treatment via High Intensity Focused Ultrasound in a surgical center demonstrated that material costs (US$851.58 – SD = 2.17) had a greater impact on the total ADC compared to ADC for medications/solutions (US$72.13 – SD = 25.84)^(19)^.

A quantitative study, which aimed to estimate the Peripherally Inserted Central Catheter insertion ADC in adult patients of a Cardiopulmonary Intensive Care Unit of a public teaching hospital, measured the ADC of US$259.81 (SD ± 36.94), related to materials, and US$26.22 (SD ± 9.01), related to performing nurses’ DL^(20)^.

In the care provided by ALS teams, the ADC of DL of physicians and nurses overlapped with the ADC of materials, medications and solutions. It is important to note that, despite frequent searches in the literature, no studies were found that have determined the costs associated with the DL of healthcare professionals working in SAMU. Regarding bottom-up microcosting studies, in addition to being scarce, few show the prevalence of costs with the DL of human resources in relation to material, medication and solution costs^(21,22)^.

A quantitative, cross-sectional, analytical study, with microcosting analysis by absorption, carried out at the Human Milk Bank of a university hospital in southern Brazil, regarding the application of local and transcutaneous laser therapy by intravascular laser irradiation of blood in nipple trauma treatment, showed that, in three groups studied (control group, local laser group and intravascular laser irradiation of blood group), the cost of nurses’ DL had the greatest representation in the procedure total cost, with a variation of 76.00% to 94.00%^(21)^.

Quantitative, exploratory-descriptive research, in the form of a single case study, carried out in a Dialysis Center of a public teaching and research hospital, measured the ADC of the procedures performed to manage complications of vascular access for conventional hemodialysis. It was evident that, in most procedures, ADC with nursing professionals’ DL prevailed in the composition of the total ADC. In the administration of easily diluted antibiotics, in the administration of difficult-to-dilute antibiotics, in the administration of antibiotics without dilution, in the dressing with topical antibiotic at the central venous catheter insertion site and in arteriovenous fistula puncture, ADC with professionals was superior to ADC with materials, medications and solutions^(22)^.

In ALS care, the intraosseous puncture kit was the material that contributed most to the total direct cost. This item, despite being used in only five of the 188 care sessions, had a high unit cost (US$193.68), corresponding to 58.78% of the total for materials, medications and solutions. Despite the high unit cost, this kit, available only in the ALS vehicle, is considered a relevant item, as it provides rapid intervention in an emergency situation, when peripheral venous access is impossible, favoring the establishment of a route for administering solutions and medications^(23)^.

Macrodrops equipment, despite its low unit value (US$0.61), was the most used input in 130 of the 188 ALS care sessions, and contributed with 4.91% of the total input cost. There was greater variety in material consumption in ALS care sessions, since more invasive and more complex procedures were performed. Therefore, in a single care session, the team performed everything from immobilizations, capillary blood glucose checks and dressings to intraosseous punctures, orotracheal intubations and medication administrations. Despite this variety, it is reiterated that of the US$19.70 (SD = 36.64) of ADC per ALS care session, the cost of materials accounted for 44.24%, with ADC with DL predominating (55.76%).

The acquisition prices of materials, medications and solutions consumed in BLS and ALS services were lower than those practiced in the market, due to the acquisition process through bidding, in which the Public Administration purchases materials and/or supplies, and contracts services. However, if the Bidding and Contracts Law 14,133^(24)^ were widely implemented, the prices would be different. This new law is characterized by a series of innovations, such as the exclusion of the invitation letter and price quotation modalities, and the inclusion of a new modality: competitive dialogue. It allows the use of electronic bidding systems, which facilitates the participation of companies of all sizes and from different regions of the country. Moreover, it uses arbitration in the event of disputes and includes tie-breaking criteria in addition to price, allowing greater competitiveness among participating companies^(24)^.

It is important to note that identifying material resource costs provides nurses with specific knowledge to help strengthen the argument about the cost-benefit of their acquisition, aiming at the provision of safe and quality healthcare services as well as the successful conduct of bidding processes^(25)^.

Given the financial impact associated with material resource consumption, particularly in public services with restricted budgets, the need to improve their management is highlighted, which has been increased with nurses’ qualified participation, who have contributed to making it possible to supply materials in sufficient quality and quantity to provide safe and quality care^(25,26)^.

It is confirmed that cost management can help in the assessment of a certain type of healthcare service provision, as well as its implications for the results to be achieved. Therefore, it becomes an essential instrument in health organizations, since, with the increase in care demands, costs associated with treatments also increase. Thus, there is a need and relevance for healthcare professionals to acquire knowledge about care costs and, based on them, seek possibilities that aim to guarantee their applicability in order to enhance satisfactory results in the population’s health conditions, given the limited and restricted resources available^(27)^.

In the macroeconomic dimension, the governance of financing a care network occurs through regional action plans, with details of financial resources, the responsibility of each entity in the sustainability of the plans and the resources that will be allocated by healthcare service providers. It is operationalized through instruments and mechanisms for regulating care, such as general programming, contracting of public establishments and/or private providers and regulation of access^(28)^.

From this perspective, a case study that assessed the governance system for financing the Emergency Care Network mobile pre-hospital component in a health region in the state of Paraná showed how incipient SAMU governance is, especially in terms of financing, with little detail on financial resources, including how much the municipality receives in incentives from other entities, how much it invests of its own resources to maintain the service, as well as the lack of effective governance spaces for this network. It concludes that the deficiency in SAMU financing governance increases its difficulty in organizing and coordinating the integration of the Emergency Care Network components^(29)^.

Despite government advances in creating a specific chunk of resources linked to emergency actions, there are still considerable challenges for the full management of SAMU at all levels of government, with the need for regulation and effective integration between the other levels of care. A study that described and analyzed the evolution of federal public spending on SAMU, comparing municipalities, regions and health macro-regions in the state of Bahia between 2009 and 2012, highlighted, at the time, the need to increase funding for SAMU, greater and better distribution of resources, redressing existing inequalities between the macro-regions and health regions, as well as the essential need for greater investments in the SUS care network structure and organization, to effectively absorb demands arising from rescue, ensuring comprehensive care^(12)^.

### Implications for Practice

This study represents an advance in knowledge, as it addresses financial aspects associated with care provided by BLS and ALS teams of a SAMU. Given the lack of studies of this nature, the results obtained may help to support care and management decision-making regarding the rational allocation of healthcare professionals, materials, medications and solutions required, increasing their potential and avoiding the occurrence of waste and risks to the quality of care provided to users.

### Study Limitation

Limitations of this study include the fact that data were not collected from non-participant observations and the impossibility of covering the DL of ALS and BLS vehicle drivers, as well as the fuel consumption required by these vehicles.

## CONCLUSION

In services provided by BLS teams, ADCs with materials, medications and solutions (US$2.10) were more representative in the composition of the total ADC (US$3.21), unlike services provided by ALS teams, in which there was a predominance, in the total ADC (US$19.70), of ADC related to physicians’ and nurses’ DL (US$10.98 – SD = 5.81).

Thus, the total ADC for the sample of 380 SSRs was US$4,319.92 (100.00%), of which 85.73% referred to the 188 ALS services and 14.27% to BLS services. Extrapolating the ADC to the population of 28,267 SSRs, the total ADC for services provided by SAMU teams in 2022 would correspond to US$125,201.17 (100.00%), of which 67.11% related to 26,177 BLS services and 32.89% to 2,090 ALS services.

Identifying costs of PHC provided by BLS and ALS teams has the potential to support strategic care and management decision-making, aiming at SAMU’s financial and economic sustainability.

## Data Availability

This is a single-case study whose data set supporting the results is not publicly available due to the presence of sensitive information and the need to preserve participant confidentiality. All relevant information for understanding the findings is described in the body of the article.
