# Supplementary material for: Mobile Emergency Care Service: direct costs for personnel, materials, medications and solutions
Source: Rev Esc Enferm USP. 2025 Aug 11;59:e20250156. doi: 10.1590/1980-220X-REEUSP-2024-0156en (PMC12345813; doi:10.1590/1980-220X-REEUSP-2024-0156en)
Supplement: Translation

# Translation: Serviço de Atendimento Móvel de Urgência: custos diretos com pessoal, materiais, medicamentos e soluções*

**DOI:** 10.1590/1980-220X-REEUSP-2024-0156pt

**Authors:** Luciana Mota Leonor do Nascimento, Antônio Fernandes Costa Lima

**Affiliations:** 1 Universidade de São Paulo, Escola de Enfermagem, Programa de Pós-Graduação em Gerenciamento em Enfermagem, São Paulo, SP, Brasil.; 2 Universidade de São Paulo, Escola de Enfermagem, Departamento de Orientação Profissional, São Paulo, SP, Brasil.

**Keywords:** Assistência Pré-Hospitalar, Serviços Médicos de Emergência, Enfermagem em Emergência, Unidades Móveis de Emergência, Custos Diretos de Serviços

## Abstract

**Objetivo:**

Identificar os custos diretos médios com pessoal, materiais, medicamentos e soluções requeridos ao atendimento pré-hospitalar do Serviço de Atendimento Móvel de Urgência de um município de São Paulo.

**Método:**

Microcusteio *bottom-up* por absorção retrospectivo na modalidade de estudo de caso único*.* Coletaram-se os dados em amostra de 380 Fichas de Atendimento Sistematizado relativas a 2022. Obtiveram-se os custos multiplicando-se o tempo despendido por profissional pelo custo unitário da mão de obra direta, acrescentando-se o custo dos insumos.

**Resultados:**

Nos atendimentos das equipes de Suporte Avançado de Vida, os custos diretos médios com médicos e enfermeiros corresponderam a US$10,98, e com materiais, medicamentos e soluções, a US$8,72, totalizando US$19,70/atendimento. Nos atendimentos das equipes de Suporte Básico de Vida, os custos diretos médios com técnico de enfermagem foram de US$1,11, e com materiais, medicamentos e soluções, de US$2,10, totalizando US$3,21/atendimento.

**Conclusão:**

O custeio do atendimento pré-hospitalar móvel pode subsidiar a tomada de decisões estratégicas assistenciais e gerenciais, visando à manutenção da sua sustentabilidade financeiro-econômica.

## INTRODUÇÃO

No Brasil, os serviços de emergência pré-hospitalares móveis são considerados essenciais, pois propiciam o reconhecimento precoce de um agravo e o fornecimento das intervenções necessárias para salvar vidas, com o envio de recursos adequados, atendimento imediato e transporte para um local de referência integrado ao Sistema Único de Saúde (SUS)^(1)^.

O atendimento pré-hospitalar (APH) móvel, de urgência e emergência, tem sido cada vez mais requisitado, devido ao crescente número de acidentes, aumento da violência e ineficácia na estruturação da rede dos serviços de saúde^(2)^. Nesta conjuntura, o Serviço de Atendimento Móvel de Urgência (SAMU) oferece atendimento precoce às vítimas de agravos à saúde de qualquer natureza, diminuindo o número de óbitos e favorecendo a recuperação mais rápida com a redução de sequelas^(3)^.

O atendimento a urgências e emergências no SAMU se inicia na Central de Regulação das Urgências (CRU), onde as chamadas da população são acolhidas, há escuta qualificada e classificação de risco do paciente. A CRU aciona a unidade móvel adequada, seja para atendimento no local, por meio da equipe de Suporte Básico de Vida (SBV) ou de Suporte Avançado de Vida (SAV), ou para encaminhamento ao serviço de saúde mais apropriado ao caso. No atendimento local, a equipe avalia a vítima e realiza os primeiros socorros, seguindo as orientações do médico regulador^(3)^.

No SAMU, a equipe de SAV é composta pelo condutor de ambulância, médico clínico geral e enfermeiro, e a equipe de SBV, pelo condutor de ambulância, enfermeiro ou técnico ou auxiliar de enfermagem^(4)^. O APH móvel é pautado em protocolos desenvolvidos pelo Ministério da Saúde, juntamente com o Hospital Alemão Oswaldo Cruz, por meio do Programa de Apoio ao Desenvolvimento Institucional do SUS, o qual estabelece parcerias estratégicas impulsionando a pesquisa, a inovação, a qualificação de recursos humanos e a gestão em saúde^(5)^.

A Portaria n^o^ 2048/GM de 05/11/2002 determina as atribuições e competências dos profissionais de saúde atuantes nas equipes do SAMU, estabelecendo a seguinte composição: um médico responsável técnico, habilitado ao exercício do APH, gestor das atividades médicas; médicos reguladores, responsáveis por gerenciar a operacionalização dos atendimentos; médicos intervencionistas, responsáveis pelo atendimento do paciente, desde a reanimação à estabilização, no local da ocorrência e durante o transporte; enfermeiro, responsável pela gestão das atividades assistenciais de enfermagem; enfermeiros intervencionistas, responsáveis pelos atendimentos de enfermagem necessários à reanimação e estabilização do paciente, no local da ocorrência até o transporte; e técnicos de enfermagem, responsáveis pela assistência de nível técnico, sob a supervisão de um enfermeiro^(2)^.

Os atendimentos realizados pelas equipes do SAMU podem ser classificados como primários, quando a solicitação é feita por um usuário, e secundários, quando a solicitação é feita por um serviço de saúde, para que o paciente seja transferido para outra unidade de saúde de maior complexidade, visando à continuidade no tratamento. Independentemente da modalidade assistencial (SAV ou SBV), ressalta-se a responsabilidade das equipes ao prestar assistência ao indivíduo acometido por agravos à saúde, estabilizando e procedendo ao encaminhamento seguro e de qualidade aos equipamentos de saúde^(6)^.

Os dados gerados em cada atendimento (informações do paciente, da ocorrência e dos procedimentos executados) são registrados nas Fichas de Atendimento Sistematizado (FASis), as quais conferem respaldo legal à equipe e ao paciente, bem como visibilidade ao atendimento prestado, fundamentando análises epidemiológicas essenciais ao aprimoramento do APH local e sua continuidade^(7)^.

Em 2023, o SAMU esteve presente em 67,3% dos municípios brasileiros, atendendo 85% da população^(8)^. Seu custeio tem sido de responsabilidade tripartite entre a união, com 50% das despesas, estados e municípios, que cobrem as despesas conforme a pactuação estipulada em cada Comissão Intergestores Bipartite^(3)^.

Para a construção de Centrais de Regulação Médica de Urgências (CRMUs) e ampliação daquelas já existentes, os recursos obedecem aos seguintes parâmetros: R$216.000,00, quando a população municipal é de até 350.000 habitantes; R$350.000,00, quando a população municipal é de 350.001 a 3.000.000 habitantes; R$440.000,00, quando a população municipal é acima de 3.000.000 habitantes^(9)^. Para o custeio mensal, seguem os parâmetros: R$54.600,00 mensais, quando habilitada; e R$68.386,50 mensais, quando habilitada e qualificada de até 350.000 habitantes^(10)^.

Os valores para aquisição de equipamentos de tecnologia e informática das CRMUs abrangem até R$96.847,21, quando a população municipal é de até 350.000 habitantes; até R$288.817,15, quando a população municipal é entre 350.001 e 10.000.000 habitantes; e R$302.902.15, quando a população municipal é acima de 10.000.001 habitantes^(9)^.

Para manutenção das equipes implantadas efetivamente, foram estabelecidos os valores de R$17.062,50 mensais por unidade de SBV habilitada; R$28.494,70 mensais por unidade de SBV habilitada e qualificada; R$50.050,00 mensais por unidade de SAV habilitada; R$62.687,30 mensais por unidade de SAV habilitada e qualificada; R$54.600,00 mensais (população de até 350.000 habitantes) por unidade de CRMU habilitada; e R$68.386,50 mensais (população de até 350.000 habitantes) por unidade de CRMU habilitada e qualificada^(9)^.

O custeio mensal destinado ao SAMU está diretamente ligado aos registros mensais de atendimentos, e poderá ser suspenso quando não forem cumpridos os requisitos de habilitação e qualificação, o registro de atendimentos for inferior ao estabelecido pela portaria específica da Secretaria de Atenção à Saúde, mediante ausência de registro de atendimentos por três meses consecutivos, ou forem constatadas irregularidades no serviço^(9)^.

A gestão municipal é responsável pela dispensação de recursos materiais, humanos e financeiros necessários à viabilidade do SAMU para que não haja prejuízo da qualidade nos atendimentos prestados^(3)^. Destaca-se que os municípios foram estimulados a implementar o SAMU devido ao incentivo financeiro para a sua efetivação, além da distribuição de veículos equipados^(9)^. Estudo realizado em 2022 mostrou que o SAMU registrou 133,4 milhões de procedimentos entre os anos de 2015 e 2019. A população coberta aumentou 9,7%; o número de chamadas via 192 aumentou 26,5%; e o total de procedimentos realizados pelo SBV e SAV cresceu 28,5%^(11)^.

A política nacional de ampliação dos serviços de urgência e emergência requer um financiamento compatível com as exigências do SUS, remetendo à suficiência e distribuição adequada dos recursos^(12)^. Sabe-se que uma boa gestão é uma base estratégica para regulação e organização da assistência prestada pelo SAMU. Entretanto, existem alguns problemas no sistema de saúde que podem repercutir na qualidade dos atendimentos, como investimento inadequado, precarização de recursos, falta de infraestrutura, falta de recursos humanos e falta de manutenção das viaturas, fazendo-se necessário que as autoridades gestoras se empenhem em realizar os investimentos requeridos^(13)^.

Estudo realizado em 2022, na região do Grande ABC, em São Paulo, evidenciou que gestores ressaltaram o aumento das demandas pelo SAMU, porém sem o aumento do aporte financeiro. Salientaram, ainda, a falta de equipamentos e insumos necessários ao atendimento, reforçando que a falta de recursos poderia comprometer o alcance de resultados favoráveis^(14)^.

Tendo em vista que os recursos financeiros são limitados e que as demandas por atendimentos são crescentes, uma etapa importante para o gerenciamento de custos é a apuração dos custos associados ao APH. Assim, objetivou-se identificar os custos diretos médios (CDMs) com pessoal, materiais, medicamentos e soluções requeridos ao APH do SAMU de um município de São Paulo.

## MÉTODO

### Tipo ou Desenho do Estudo

Trata-se de microcusteio *bottom-up* por absorção retrospectivo na modalidade de estudo de caso único*.*

### Local

A pesquisa foi conduzida na Base Regional do SAMU de um município brasileiro do estado de São Paulo. Em 2022, o referido SAMU recebeu 85.709 chamados (75.429 clínicos, 5.964 de trauma, 1.202 obstétricos, 1.137 pediátricos e 1.977 psiquiátricos), os quais resultaram em 28.267 atendimentos *in loco*, sendo 26.177 de SBV e 2.090 de SAV.

### Amostra e Critérios de Seleção

A partir da população de FASis (N=28.267), referente ao ano de 2022, um estatístico calculou o quantitativo amostral mínimo a ser analisado, utilizando-se a metodologia de dimensionamento amostral para a estimativa de uma média. Como não havia uma estimativa de desvio padrão (DP) do custo, assumiu-se a margem de erro ε = 0,1σ, isto é, de no máximo 10% do DP. Assim, obteve-se o cálculo amostral de 380 FASis, 188 relacionadas aos atendimentos prestados por equipes de SAV e 192 por equipes de SBV.

Foram selecionadas as FASis que estavam adequadamente preenchidas com as variáveis de identificação do chamado (município, data, horário, viatura acionada) e do paciente (sexo e idade), local da ocorrência, tempo despendido e insumos (materiais, medicamentos e soluções) consumidos. Foram excluídas as FASis cujo preenchimento estivesse incompleto.

### Coleta de Dados

Todos os dados, inclusive os relativos ao tempo despendido nos atendimentos prestados pelos profissionais de saúde, foram coletados de maio a agosto de 2023 por intermédio do acesso eletrônico à amostra de FASis preenchidas pelas equipes de SBV e SAV na sala da Coordenação Administrativa da Secretaria de Saúde Municipal.

O estudo consistiu em microcusteio *bottom-up* por absorção, no qual houve a consulta dos recursos reais consumidos por um paciente ou serviço de saúde, determinando-se o custo real de ofertar cuidados de saúde por um prestador. Optou-se pelo método de microcusteio *bottom-up* por permitir definir, detalhadamente, todas as variáveis componentes dos custos, a partir de dados individuais do tratamento destinado ao paciente, com a revisão de prontuários ou de ficha clínica específica para o estudo^(15)^.

O custeio por absorção corresponde à apropriação dos custos de produção aos bens produzidos. Desse modo, todos os gastos referentes à produção são compartilhados entre os serviços ou produtos realizados; por meio dele, designam-se os custos de fabricação ao custo do produto^(16^.

Assim, calcularam-se os custos diretos, definidos como aqueles que podem ser identificados e quantificados, os quais referem-se a um valor monetário relativo ao consumo de mão de obra direta (MOD), materiais, medicamentos e soluções^(16^. A MOD refere-se aos profissionais que trabalham sobre um determinado produto/serviço, desde que seja possível mensurar o tempo consumido à sua realização. É composta pelo salário, encargos, férias e 13º salário^(16^.

Calculou-se a MOD dos profissionais de saúde a partir de informações salariais fornecidas pelo Departamento de Recursos Humanos (DRH) municipal responsável pelo SAMU campo de estudo. Para o custeio, os valores, obtidos em reais (R$), foram convertidos para o dólar americano (US$) pela taxa média de US$4,75/R$1,00, com base nas cotações de janeiro a dezembro de 2022 fornecidas pelo Banco Central do Brasil.

Assim, a média ponderada da massa salarial do médico correspondeu a US$252,63/12 horas, US$21,05/hora e US$0,35/minuto; do enfermeiro correspondeu a US$105,26/12 horas, US$8,77/hora e US$0,15/minuto; e do técnico de enfermagem correspondeu a US$52,63/12horas, US$4,38/hora e US$0,07/minuto. As informações relativas aos custos das últimas aquisições de insumos materiais, medicamentos e soluções foram disponibilizadas pelo Departamento de Compras/Almoxarifado.

Esclarece-se que, no período de realização do estudo, a condução das viaturas de SAV e SBV consistia em uma atividade delegada a bombeiros militares remunerados pelo Estado, cujas informações salariais não eram de domínio do DRH supracitado. Não foi possível custear o consumo de combustível das viaturas, tendo em vista que todos os veículos do município eram abastecidos no mesmo local e o pagamento era efetuado de forma única, não sendo possível distinguir as viaturas de outros veículos.

Para obter o CDM dos atendimentos registrados pelas equipes de SAV e de SBV, utilizou-se a equação^(17)^ $\bar{C\left(P_{\text{τ}}\right)}=\sum_{k=1}^{n}\left(\bar{q_{k}.}\;\bar{Pu_{k}}\right)+\sum_{k=1}^{n}\left(\bar{qs_{k}.}\;\bar{Psu_{k}}\right)+\sum_{c=1}^{n}\left(\bar{t_{c}.}\;\bar{su_{c}}\right)$ , abrangendo as seguintes variáveis: $\bar{qm_{k}}$ (quantidade média de materiais); $\bar{Pmu_{k}}$ (preço unitário de cada material); $\bar{qs_{k}}$ (quantidade média de soluções/medicamentos); $\bar{Psu_{k}}$ (preço unitário de cada solução/medicamento); $\bar{t_{c}}$ (tempo médio despendido por cada profissional); $\bar{Su_{c}}$ (massa salarial unitária de cada mão de obra)^(17)^. Em síntese, multiplicou-se o tempo despendido pelo profissional executante, de acordo com a categoria, pelo custo da respectiva MOD, acrescido do custo dos materiais, medicamentos e soluções^(17)^ consumidos em cada atendimento de SAV e SBV.

### Análise e Tratamento dos Dados

Organizaram-se os dados em planilha eletrônica, por meio de digitação dupla independente, os quais foram tratados por intermédio de estatística descritiva e inferencial.

### Aspectos Éticos

O Comitê de Ética em Pesquisa da instituição proponente aprovou, após a anuência da Secretaria de Saúde Municipal responsável pelo SAMU estudado, a realização do estudo (Parecer n^o^ 6.031.451, de 28 de abril de 2023).

## RESULTADOS

A partir da amostra de 192 FASis referentes aos atendimentos das equipes do SBV, obteve-se o tempo médio de atendimento do técnico de enfermagem de 15,85 minutos. Indica-se, na Tabela 1, que o CDM total correspondeu a US$3,21/atendimento (DP = 2,61), com maior representatividade do CDM com materiais, medicamentos e soluções (US$2,10–DP = 2,26).

**Tabela 1:** Distribuição dos custos em dólar (US$*) da mão de obra direta dos técnicos de enfermagem atuantes nos 192 atendimentos das equipes do Suporte Básico de Vida segundo a média, desvio padrão, mínimo, máximo, 1º quartil, mediana e 3º quartil – São Paulo, SP, Brasil, 2023.

Variáveis	CDM (US$*)	Desvio padrão	Porcentagem de composição do CDM total (%)	Mínimo (US$*)	Máximo (US$*)	1^o^ quartil (US$)	Mediana (US$)	3^o^ quartil (US$)
**CDM total com MOD de técnico de enfermagem**	**1,11**	**0,75**	**34,55%**	0,15	4,82	0,58	0,95	1,41
**CDM total com materiais, medicamentos e soluções**	**2,10**	**2,26**	**65,45%**	0,00	18,18	0,41	1,68	3,05
**CDM total por atendimento de SBV**	**3,21**	**2,61**	**100,00%**	0,15	19,94	0,34	2,39	4,40

Nota: *Taxa média de conversão: R$4,75/US$1,00. Banco Central, com base na cotação de janeiro a dezembro de 2022; MOD – mão de obra direta; SBV – Suporte Básico de Vida; CDM – custo direto médio.

De acordo com os registros dos materiais, medicamentos e soluções constantes das 192 FASis analisadas, o custo total dos atendimentos de SBV foi de US$396,67 (100,00%), com CDM de US$2,10 por atendimento. Na Tabela 2, evidencia-se que, entre 39 itens, os que mais contribuíram para a composição deste custo total foram colar cervical P/33 atendimentos (custo unitário: US$3,00; 24,94%), solução fisiológica 0,9% 250 ml/40 atendimentos (custo unitário: US$0,93; 9,19%), tala M/14 atendimentos (custo unitário: US$2,57; 9,07%), e colar cervical M/10 atendimentos (custo unitário: US$3,19; 8,04%).

**Tabela 2:** Distribuição dos custos em dólar (US$*) dos materiais, medicamentos e soluções consumidos nos 192 atendimentos do Suporte Básico de Vida segundo o custo total, porcentagem de composição do custo total, quantitativo de atendimentos, custo direto médio por atendimento e valor unitário – São Paulo, SP, Brasil, 2023.

Materiais, medicamentos e soluções (insumos)	Custo total (US$)	Porcentagem de composição do custo total (%)	Quantitativo de atendimentos	CDM do insumo por atendimento (US$)	Custo unitário do insumo (US$)
Colar cervical P	98,93	24,94	33	3,00	3,00
Solução fisiológica 0,9% 250 ml	36,45	9,19	40	0,93	0,93
Tala aramada M	35,99	9,07	14	2,57	2,57
Colar cervical M	31,89	8,04	10	3,19	3,19
Equipo macrogotas	22,05	5,56	38	0,61	0,61
Máscara de alta concentração	20,77	5,24	8	2,60	2,60
Tala aramada – G	18,91	4,77	5	3,78	3,78
Solução fisiológica 0,9% 500 ml	16,45	4,15	17	0,97	1,10
Tala aramada – P	15,24	3,84	6	2,54	2,18
Atadura 12 cm	13,60	3,43	53	0,26	0,16
Fita para glicemia capilar	13,41	3,38	93	0,15	0,15
Gaze algodonada	11,76	2,96	12	0,98	0,73
Tala aramada – GG	8,11	2,05	1	8,11	4,06
Cateter não agulhado 20	7,12	1,80	18	0,42	0,42
Cateter não agulhado 22	6,25	1,58	15	0,45	0,45
Cateter nasal tipo óculos	5,46	1,38	32	0,17	0,17
Luva de procedimento – P (látex/com pó)	4,71	1,19	89	0,05	0,03
Glicose 50% ampola 10 ml	4,28	1,08	12	0,36	0,12
Gaze estéril (pacote)	3,82	0,96	27	0,14	0,14
Luva de procedimento – M (látex/com pó)	3,69	0,93	74	0,03	0,03
Lanceta para glicemia capilar	3,07	0,77	93	0,03	0,03
Bandagem triangular – M	2,88	0,73	4	0,72	0,72
Cateter agulhado 21	2,15	0,54	5	0,43	0,43
Cateter agulhado 23	2,09	0,53	5	0,42	0,42
Cateter não agulhado 18	1,89	0,48	4	0,47	0,47
Luva de procedimento – G (látex/com pó)	1,30	0,33	28	0,05	0,02
Seringa 20 ml	1,15	0,29	14	0,07	0,07
Cateter não agulhado 14	0,95	0,24	1	0,95	0,95
Cateter não agulhado 24	0,49	0,12	1	0,49	0,49
Cânula de Guedel 4	0,44	0,11	1	0,44	0,44
Dipirona sódica 500 mg ampola	0,23	0,06	1	0,23	0,23
Agulha 40 × 12	0,20	0,05	15	0,01	0,01
Máscara N95	0,20	0,05	1	0,20	0,20
Eletrodos para monitorização	0,20	0,05	1	0,20	0,04
Butilbrometo de escopolamina ampola	0,20	0,05	1	0,20	0,20
Seringa 10 ml	0,12	0,03	2	0,06	0,04
Captopril 25 mg comprimido	0,12	0,03	4	0,02	0,02
Água destilada ampola 10 ml	0,11	0,03	1	0,11	0,05
Macacão descartável	0,01	0,00	1	0,01	0,01

Nota: *Taxa média de conversão: R$4,75/US$1,00. Banco Central, com base na cotação de janeiro a dezembro de 2022; MOD – mão de obra direta; CDM – custo direto médio.

Considerando a amostra de 188 FASis relativas aos atendimentos das equipes do SAV, verifica-se, na Tabela 3, que o CDM total correspondeu a US$19,70/atendimento (DP = 36,34), com maior representatividade do CDM total com MOD dos profissionais de saúde (US$10,99–DP = 5,81), cujo tempo médio de atendimento conjunto correspondeu a 22 minutos.

**Tabela 3:** Distribuição dos custos em dólar (US$*) da mão de obra direta dos profissionais de saúde atuantes nos 188 atendimentos do Suporte Avançado de Vida e dos insumos consumidos segundo a média, desvio padrão, mínimo, máximo, 1º quartil, mediana e 3º quartil – São Paulo, SP, Brasil, 2023.

Variáveis	CDM (US$*)	Desvio padrão	Porcentagem de composição do CDM total (%)	Mínimo(US$*)	Máximo(US$*)	1^o^ quartil (US$)	Mediana (US$)	3^o^ quartil (US$)
CDM com MOD de enfermeiro	3,23	2,12	16,40	0,58	15,79	1,90	3,80	3,80
CDM com MOD de médico	7,75	5,10	39,36	1,40	37,89	4,56	6,67	9,12
**CDM total com MOD**	**10,99**	**5,81**	**55,76**	1,99	53,68	6,46	9,44	12,92
**CDM total com materiais, medicamentos e soluções**	**8,72**	**32,06**	**44,24**	0,27	212,46	2,24	3,02	5,12
**CDM total de atendimento por SAV**	**19,70**	**36,64**	**100,00**	**2,79**	**256,51**	**2,24**	**13,27**	**18,05**

Nota: *Taxa média de conversão: R$4,75/US$1,00. Banco Central, com base na cotação de janeiro a dezembro de 2022; MOD – mão de obra direta; SAV – Suporte Avançado de Vida; CDM – custo direto médio.

Segundo os registros dos materiais, medicamentos e soluções constantes das 188 FASis, o custo total correspondeu a US$1.647,49 (100,00%), com um CDM de US$8,72 por atendimento. Conforme a Tabela 4, entre os 73 itens consumidos, os que mais contribuíram para a composição deste custo total foram *kit* de punção intraóssea/cinco atendimentos (custo unitário: US$193,68; 58,78%), solução fisiológica 0,9%–500 ml/75 atendimentos (custo unitário: US$1,10; 5,33%), colar cervical P/27 atendimentos (custo unitário: US$3,00; 4,91%), equipo macrogotas/130 atendimentos (custo unitário: US$0,61; 4,91%) e máscara de alta concentração/21 atendimentos (custo unitário: US$2,60; 3,31%).

**Tabela 4:** Distribuição dos custos em dólar (US$) dos materiais, medicamentos e soluções consumidos nos 188 atendimentos do Suporte Avançado de Vida segundo o custo total, porcentagem de composição do custo total, quantitativo de atendimentos, custo direto médio por atendimento e valor unitário – São Paulo, SP, Brasil, 2023

Materiais, medicamentos e soluções (insumos)	Custo total (US$)	Porcentagem de composição do custo total (%)	Quantitativo de atendimentos	CDM do insumo por atendimento (US$)	Custo unitário do insumo (US$)
Kit de punção intraóssea	968,42	58,78	5	193,68	193,68
Solução fisiológica 0,9% 500 ml	87,75	5,33	75	1,17	1,10
Colar cervical P	80,94	4,91	27	3,00	3,00
Equipo macrogotas	80,87	4,91	130	0,61	0,61
Máscara de alta concentração	54,51	3,31	21	2,60	2,60
Solução ringer lactato 500 ml	38,50	2,34	22	1,75	1,60
Solução fisiológica 0,9% 250 ml	35,52	2,16	35	1,01	0,93
Tala aramada - M	23,13	1,40	8	2,89	2,57
Eletrodos para monitorização	20,60	1,25	103	0,20	0,04
Cateter não agulhado 20	20,11	1,22	48	0,42	0,42
Gaze algodonada	19,10	1,16	16	1,19	0,73
Tala aramada - G	18,91	1,15%	4	4,73	3,78
Cateter não agulhado 18	15,09	0,92	32	0,47	0,47
Cateter não agulhado 22	13,39	0,81	30	0,45	0,45
Solução glicosada 5% 250 ml	10,93	0,66	9	1,21	1,09
Atadura 12 cm	9,12	0,55	32	0,29	0,16
Cateter não agulhado 16	8,70	0,53	13	0,67	0,67
Fita para glicemia capilar	8,55	0,52	58	0,15	0,15
Luva de procedimento M (látex/com pó)	8,24	0,50	108	0,08	0,03
Epinefrina 1 mg ampola	7,52	0,46	9	0,84	0,21
Luva de procedimento - P (látex/com pó)	6,95	0,42	89	0,08	0,03
Tala aramada P	6,53	0,40	3	2,18	2,18
Tubo orotraqueal 7,5	6,46	0,39	9	0,72	0,72
Glicose 50% ampola 10 ml	6,25	0,38	14	0,45	0,12
Cloreto de suxametônio 100 mg ampola	5,91	0,36	2	2,95	2,95
Midazolam 15 mg ampola	4,21	0,26	7	0,60	0,60
Midazolam 5 mg ampola	4,04	0,25	5	0,81	0,40
Luva de procedimento G (látex/com pó)	3,98	0,24	64	0,06	0,02
Dipirona sódica 500 mg ampola	3,83	0,23	17	0,23	0,23
Etomidato ampola 10 ml	3,83	0,23	2	1,91	1,91
Cateter nasal tipo óculos	3,75	0,23	22	0,17	0,17
Cateter não agulhado 24	3,45	0,21	7	0,49	0,49
Ondansetrona ampola 2 ml	3,27	0,20	8	0,41	0,41
Cloridrato de tramadol ampola	3,23	0,20	7	0,46	0,46
Colar cervical M	3,19	0,19	1	3,19	3,19
Haloperidol 5 mg ampola	3,09	0,19	8	0,39	0,34
Cetoprofeno frasco ampola	2,77	0,17	4	0,69	0,69
Gaze estéril (pacote)	2,68	0,16	14	0,10	0,10
Cateter agulhado 23	2,51	0,15	6	0,42	0,42
Flumazenil 0,5 mg ampola	2,37	0,14	2	1,19	1,19
Omeprazol 40 mg ampola	2,27	0,14	2	1,14	1,14
Bandagem triangular – M	2,16	0,13	3	0,72	0,72
Cateter agulhado 21	2,15	0,13	5	0,43	0,43
Diazepan ampola	2,15	0,13	10	0,21	0,21
Tubo orotraqueal 8,0	2,15	0,12	3	0,67	0,67
Lanceta para glicemia capilar	1,95	0,12	58	0,03	0,03
Cateter não agulhado 14	1,89	0,12	2	0,95	0,95
Morfina ampola	1,83	0,11	2	0,92	0,92
Citrato de fentanila 50 mcg ampola	1,81	0,11	4	0,45	0,45
Clopidogrel 75 mg comprimido	1,77	0,11	6	0,29	0,07
Fenitoína ampola	1,52	0,09	3	1,03	0,60
Amiodarona 50 mg ampola	1,41	0,09	2	0,71	0,47
Seringa 5 ml	1,37	0,08	33	0,04	0,03
Cânula de Guedel 4	1,33	0,08	3	0,44	0,44
Seringa 10 ml	1,28	0,08	30	0,04	0,04
Seringa 20 ml	1,28	0,08	15	0,09	0,07
Cloridrato de prometazina ampola	1,14	0,07	3	0,38	0,38
Agulha 40 × 12	0,92	0,06	69	0,01	0,01
Água destilada ampola 10 ml	0,89	0,05	13	0,07	0,05
Ácido tranexâmico ampola	0,86	0,05	1	0,86	0,86
Conector multivias	0,77	0,05	3	0,26	0,26
Manta térmica – G	0,49	0,03	1	0,49	0,49
Furosemida ampola	0,47	0,03	1	0,47	0,24
Butilbrometo de escopolamina ampola	0,40	0,02	2	0,20	0,20
Agulha 25 × 7	0,34	0,02	13	0,03	0,02
Máscara N95	0,20	0,01	1	0,20	0,20
Captopril 25 mg comp.	0,19	0,01	6	0,03	0,02
Ácido acetilsalicílico 100 mg comprimido	0,18	0,01	8	0,02	0,01
Sonda de aspiração traqueal 10	0,12	0,01	1	0,12	0,12
Diazepan 10 mg comprimido	0,07	0,00	4	0,02	0,02
Isossorbida 5 mg comprimido	0,04	0,00	3	0,01	0,01
Macacão descartável	0,03	0,00	2	0,01	0,01
Hidroclorotiazida comprimido	0,01	0,00	1	0,01	0,01

Nota: *Taxa média de conversão: R$4,75/US$1,00. Banco Central, com base na cotação de janeiro a dezembro de 2022; MOD – mão de obra direta; SAV – Suporte Avançado de Vida; CDM – custo direto médio.

Os CDM totais referentes à amostra 380 FASis corresponderam a US$4.319,92, sendo US$616,32 relativos a 192 atendimentos de SBV, e US$3.703,60, a 188 atendimentos de SAV. Extrapolando-se os CDM para a população de 28.267 FASis, os CDM totais corresponderiam, no ano de 2022, a US$125.201,17 (100,00%), sendo US$84.028,17 (67,11%) decorrentes de 26.177 atendimentos de SBV, e US$41.173,00 (32,89%), e de 2.090 de atendimentos de SAV.

## DISCUSSÃO

Nos atendimentos das equipes de SBV, verificou-se que o CDM com materiais, medicamentos e soluções foi superior ao CDM com a MOD dos técnicos de enfermagem, contribuindo com 65,45% do CDM total por atendimento. Os materiais mais utilizados foram lanceta e fita para glicemia capilar, consumidos em 93 dos 192 atendimentos. Pesquisas recentes, abrangendo diferentes objetos de estudo, também indicaram o predomínio dos custos relacionados aos recursos materiais^(18–20)^.

Estudo de caso único, quantitativo, exploratório-descritivo, ao calcular o CDM da inserção do cateter venoso central de longa permanência em pacientes que realizavam hemodiálise em um hospital público do estado de São Paulo, obteve o CDM de US$107,01 (DP = 0,23) com material, US$22,10 (DP = 3,63) com a MOD dos profissionais, US$4,65 (DP = 0,00) com medicamentos e US$0,80 (DP = 0,15) com soluções^(18)^.

Outra pesquisa quantitativa, exploratório-descritiva, do tipo estudo de caso único, que calculou o CDM do tratamento de câncer de próstata via *High Intensity Focused Ultrasound*, em centro cirúrgico, demonstrou que os custos com materiais (US$851,58 – DP = 2,17) obtiveram maior repercussão no CDM total em comparação aos CDM de medicamentos/soluções (US$72,13 – DP= 25,84)^(19)^.

Estudo quantitativo, que objetivou estimar o CDM da inserção do Cateter Central de Inserção Periférica em pacientes adultos de uma Unidade de Terapia Intensiva Cardiopulmonar de um hospital de ensino público, mensurou o CDM de US$259,81 (DP ± 36,94), relativo aos materiais, e de US$26,22 (DP ± 9,01), relativo à MOD dos enfermeiros executantes^(20)^.

Nos atendimentos das equipes de SAV, os CDM da MOD dos médicos e enfermeiros se sobrepuseram aos CDM dos materiais, medicamentos e soluções. Esclarece-se que, apesar de buscas frequentes na literatura, não se encontraram pesquisas que tenham apurado os custos associados à MOD dos profissionais de saúde atuantes em SAMU. Com relação aos estudos de microcusteio *bottom-up*, além de serem escassos, poucos evidenciam a prevalência dos custos com a MOD de recursos humanos em relação aos custos de materiais, medicamentos e soluções^(21,22)^.

Estudo quantitativo, transversal, analítico, com análise de microcusteio por absorção, realizado no Banco de Leite Humano de hospital universitário do sul do Brasil, referente à aplicação de laserterapia local e transcutânea por *intravascular laser irradiation of blood* no tratamento de traumas mamilares, mostrou que, em três grupos estudados (grupo controle, grupo laser local e grupo *intravascular laser irradiation of blood*), o custo com a MOD do enfermeiro teve maior representatividade no custo total do procedimento, com variação de 76,00% a 94,00%^(21)^.

Pesquisa quantitativa, exploratório-descritiva, na modalidade de estudo de caso único, realizada em um Centro de Diálise de hospital público de ensino e pesquisa, mensurou o CDM dos procedimentos realizados para manejo das complicações do acesso vascular para hemodiálise convencional. Evidenciou-se que, na maioria dos procedimentos, o CDM com MOD dos profissionais de enfermagem prevaleceu na composição do CDM total. Na administração de antibióticos de fácil diluição, na administração de antibióticos de difícil diluição, na administração de antibióticos sem diluição, no curativo com antibiótico tópico no local de inserção do cateter venoso central e na punção de fístula arteriovenosa, o CDM com profissionais foi superior ao CDM com materiais, medicamentos e soluções^(22)^.

Nos atendimentos de SAV, o *kit* de punção intraóssea foi o material que mais contribuiu para o custo direto total. Este item, apesar de utilizado em apenas cinco dos 188 atendimentos, apresentou um custo unitário elevado (US$193,68), correspondendo a 58,78% do total com materiais, medicamentos e soluções. Apesar do custo unitário elevado, esse *kit*, disponível somente na viatura de SAV, é considerado um item relevante, ao propiciar rápida intervenção em uma situação de emergência, quando o acesso venoso periférico está impossibilitado, favorecendo o estabelecimento de uma via de administração de soluções e medicamentos^(23)^.

O equipo macrogotas, apesar de seu baixo valor unitário (US$0,61), foi o insumo mais utilizado em 130 dos 188 atendimentos de SAV, e contribuiu com 4,91% do custo total dos insumos. Houve maior variedade no consumo dos materiais nos atendimentos de SAV, por serem realizados procedimentos mais invasivos e de maior complexidade. Então, em um único atendimento, a equipe realizou desde imobilizações, verificações de glicemia capilar e curativos até punçoes intraósseas, intubações orotraqueais e administrações de medicamentos. Apesar dessa variedade, reitera-se que dos US$19,70 (DP = 36,64) de CDM por atendimento de SAV, o custo com os materiais foi responsável por 44,24%, predominando o CDM com MOD (55,76%).

Os preços de aquisição dos materiais, medicamentos e soluções consumidos nos atendimentos de SBV e SAV foram menores do que os praticados no mercado, devido ao processo de aquisição por meio de licitação, no qual a Gestão Pública compra materiais e/ou insumos, e contrata serviços. Entretanto, caso a Lei de Licitações e Contratos nº 14.133^(24)^ estivesse amplamente implantada, os preços seriam diferentes. Esta nova lei caracteriza-se por uma série de inovações, como a exclusão das modalidades de carta convite e tomada de preços, e a inclusão de uma nova modalidade: o diálogo competitivo. Possibilita a utilização de sistemas eletrônicos de licitação, o que facilita a participação de empresas de todos os tamanhos e de diferentes regiões do país. Além disso, recorre à arbitragem, em caso de disputas, e inclui critérios de desempate, além do preço, permitindo maior competitividade entre as empresas participantes^(24)^.

Ressalta-se que a identificação dos custos dos recursos materiais fornece aos enfermeiros conhecimentos específicos para ajudar no fortalecimento da argumentação sobre o custo-benefício de sua aquisição, visando à prestação de serviços de saúde seguros e de qualidade, bem como à condução exitosa de processos licitatórios^(25)^.

Mediante a repercussão financeira associada ao consumo de recursos materiais, notadamente nos serviços públicos com orçamentos restritos, ressalta-se a imprescindibilidade de aperfeiçoar o seu gerenciamento, o qual vem sendo incrementado com a participação qualificada dos enfermeiros, que têm contribuído para viabilizar o fornecimento de materiais em qualidade e quantidade suficientes para a prestação da assistência segura e com qualidade^(25,26)^.

Corrobora-se que o gerenciamento dos custos pode auxiliar na avaliação de determinada modalidade de prestação de serviço de saúde, bem como sua implicação nos resultados a serem alcançados. Portanto, torna-se um instrumento essencial nas organizações de saúde, visto que, com o aumento das demandas assistenciais, aumentam também os custos associados aos tratamentos. Assim, estão postas a necessidade e a relevância dos profissionais de saúde se apropriarem do conhecimento sobre os custos assistenciais e, a partir deles, buscarem possibilidades que visem garantir a sua aplicabilidade de forma a potencializar os resultados satisfatórios nas condições de saúde da população, diante dos limitados e restritos recursos disponíveis^(27)^.

Na dimensão macroeconômica, a governança de financiamento de uma rede de atenção ocorre por intermédio dos planos de ação regionais, com detalhamento dos recursos financeiros, da responsabilidade de cada ente na sustentabilidade dos planos e dos recursos que serão alocados por prestadores de serviços de saúde. Operacionaliza-se por meio de instrumentos e mecanismos de regulação da atenção, como programação geral, contratualização de estabelecimentos públicos e/ou prestadores privados e regulação do acesso^(28)^.

A partir dessa perspectiva, estudo de caso que avaliou o sistema governança de financiamento do componente pré-hospitalar móvel da Rede de Atenção às Urgências e Emergências, em uma região de saúde do estado do Paraná, inferiu o quanto é incipiente a governança do SAMU, especialmente a de financiamento, com pouco detalhamento dos recursos financeiros, incluindo quanto o município recebe de incentivo dos demais entes, o quanto ele investe de recursos próprios para manutenção do serviço, bem como a não efetivação dos espaços de governança desta rede. Conclui que a deficiência na governança de financiamento do SAMU amplia sua dificuldade em ordenar e articular a integração dos componentes da Rede de Atenção às Urgências e Emergências^(29)^.

Apesar dos avanços governamentais relativos à criação de um bloco específico de recursos vinculados às ações de urgência, ainda existem desafios consideráveis para a gestão plena do SAMU em todos os níveis de governo, com a necessidade de regulação e integração efetiva entre os demais níveis de atenção. Estudo que descreveu e analisou a evolução do gasto público federal com o SAMU, comparando municípios, regiões e macrorregiões de saúde no estado da Bahia entre 2009 e 2012, evidenciou, à época, a necessidade da ampliação do financiamento destinado ao SAMU, maior e melhor distribuição de recursos, reparando as desigualdades existentes entre as macrorregiões e regiões de saúde, bem como a imprescindibilidade de maiores investimentos na estrutura e organização da rede assistencial do SUS, para absorver, resolutivamente, as demandas provenientes do resgate, assegurando a integralidade da atenção^(12)^.

### Implicações Para a Prática

Este estudo representa um avanço do conhecimento, ao tratar de aspectos financeiros associados aos atendimentos realizados por equipes de SBV e SAV de um SAMU. Diante da ausência de estudos desta natureza, os resultados obtidos poderão auxiliar na fundamentação de tomada de decisões assistenciais e gerenciais quanto à alocação racional dos profissionais de saúde, materiais, medicamentos e soluções requeridos, incrementando a sua potencialidade e evitando a ocorrência de desperdícios e riscos à qualidade da assistência prestada ao usuário.

### Limitação do Estudo

Como limitações deste estudo, indicam-se não terem sido coletados os dados a partir de observações não participantes e a impossibilidade de custear a MOD dos condutores das viaturas de SAV e SBV, bem como do consumo de combustível requerido por essas viaturas.

## CONCLUSÃO

Nos atendimentos das equipes de SBV, os CDM com materiais, medicamentos e soluções (US$2,10) foram mais representativos na composição do CDM total (US$3,21), diferentemente dos serviços prestados pelas equipes SAV, em que houve predominância, no total de CDM (US$19,70), de CDM referente ao MOD de médicos e enfermeiros (US$10,98 –DP = 5,81).

Assim, obteve-se o CDM total da amostra de 380 FASis de US$4.319,92 (100,00%), sendo 85,73% referentes aos 188 atendimentos de SAV, e 14,27%, aos atendimentos do SBV. Extrapolando os CDM para a população de 28.267 FASis, os CDMs totais de atendimentos, em 2022, pelas equipes do SAMU, corresponderiam a US$125.201,17 (100,00%), sendo 67,11% relativos a 26.177 atendimentos de SBV e, 32,89%, a 2.090 de atendimentos de SAV.

A identificação dos custos dos APH prestado por equipes de SBV e SAV tem potencial para subsidiar a tomada de decisões assistenciais e gerenciais estratégicas, visando à sustentabilidade financeiro-econômica do SAMU.

## Data Availability

Trata-se de um estudo de caso único cujo conjunto de dados que dá suporte aos resultados não está disponível publicamente devido à presença de informações sensíveis e à necessidade de preservação da confidencialidade dos participantes. Todas as informações relevantes para a compreensão dos achados estão descritas no corpo do artigo.

# Translation: Servicio Móvil de Atención de Urgencias: costes directos de personal, material, medicamentos y soluciones*

**DOI:** 10.1590/1980-220X-REEUSP-2024-0156eS

**Authors:** Luciana Mota Leonor do Nascimento, Antônio Fernandes Costa Lima

**Affiliations:** 1 Universidade de São Paulo, Escola de Enfermagem, Programa de Pós-Graduação em Gerenciamento em Enfermagem, São Paulo, SP, Brasil.; 2 Universidade de São Paulo, Escola de Enfermagem, Departamento de Orientação Profissional, São Paulo, SP, Brasil.

**Keywords:** Atención Prehospitalaria, Servicios Médicos de Urgencia, Enfermería de Urgencia, Ambulancias, Costos Directos de Servicios

## Abstract

**Objetivo:**

Identificar los costos directos promedio de personal, materiales, medicamentos y soluciones requeridos para la atención prehospitalaria del Servicio Móvil de Urgencias de una ciudad de São Paulo.

**Método:**

Microcosteo bottom-up de absorción retrospectivo mediante un estudio de caso único. Los datos se recopilaron de una muestra de 380 Historiales de Atención Sistematizados de 2022. Los costos se obtuvieron multiplicando el tiempo dedicado por cada profesional por el costo unitario de la mano de obra directa, sumando el costo de los suministros.

**Resultados:**

En los servicios del equipo de Soporte Vital Avanzado, el costo directo promedio para médicos y enfermeras fue de US$10,98, y para materiales, medicamentos y soluciones, de US$8,72, totalizando US$19,70 por servicio. En los equipos de Soporte Vital Básico, el costo directo promedio para técnicos de enfermería fue de US$1,11, y para materiales, medicamentos y soluciones, de US$2,10, totalizando US$3,21 por servicio.

**Conclusión:**

El costo de la atención prehospitalaria móvil puede respaldar la toma de decisiones estratégicas de atención y gestión, con el objetivo de mantener su sostenibilidad financiera y económica.

## INTRODUCCIÓN

En Brasil, los servicios móviles de emergencia prehospitalaria se consideran esenciales, ya que proporcionan el reconocimiento precoz de una lesión y las intervenciones necesarias para salvar vidas, con el envío de los recursos adecuados, la atención inmediata y el transporte a un lugar de referencia integrado en el Sistema Único de Salud (SUS)^(1)^.

La asistencia prehospitalaria móvil de urgencia y emergencia (APH) viene siendo cada vez más demandada debido al creciente número de accidentes, al aumento de la violencia y a la ineficacia en la estructuración de la red de servicios sanitarios^(2)^. En este contexto, el Servicio de Atención Móvil de Urgencia (SAMU) presta atención precoz a las víctimas de problemas de salud de cualquier tipo, reduciendo el número de muertes y favoreciendo una recuperación más rápida con reducción de secuelas^(3)^.

La atención urgente y de emergencia del SAMU comienza en el Centro de Regulación de Urgencias (CRU), donde se reciben las llamadas de los ciudadanos, se realiza una escucha cualificada y se clasifica el riesgo del paciente. El CRU activa la unidad móvil adecuada, bien para la atención in situ por el equipo de Soporte Vital Básico (SVB) o Avanzado (SVA), bien para la derivación al servicio sanitario más apropiado. En el servicio local, el equipo evalúa a la víctima y le presta los primeros auxilios, siguiendo las directrices del regulador médico^(3)^.

En el SAMU, el equipo de SVA está formado por el conductor de la ambulancia, el médico de cabecera y la enfermera, y el equipo de SVB está formado por el conductor de la ambulancia, la enfermera o el técnico o auxiliar de enfermería^(4)^. La APH móvil se basa en protocolos desarrollados por el Ministerio de Salud, junto con el Hospital Alemán Oswaldo Cruz, a través del Programa de Apoyo al Desarrollo Institucional del SUS, que establece asociaciones estratégicas que impulsan la investigación, la innovación, la cualificación de recursos humanos y la gestión sanitaria^(5)^.

La Ordenanza 2048/GM del 05/11/2002 determina las funciones y competencias de los profesionales sanitarios que trabajan en los equipos SAMU, estableciendo la siguiente composición un médico responsable, habilitado para el ejercicio de la APH, que dirige las actividades médicas; médicos reguladores, responsables de la gestión de la operacionalización de los cuidados; médicos intervencionistas, responsables de los cuidados al paciente, desde la reanimación hasta la estabilización, en el lugar de los hechos y durante el transporte; enfermeros, responsables de la gestión de los cuidados de enfermería; enfermeros intervencionistas, responsables de los cuidados de enfermería necesarios para reanimar y estabilizar al paciente, en el lugar y durante el transporte; y técnicos de enfermería, responsables de los cuidados técnicos, bajo la supervisión de un enfermero^(2)^.

La asistencia prestada por los equipos del SAMU puede clasificarse en primaria, cuando la solicitud la realiza un usuario, y secundaria, cuando la realiza un servicio sanitario, para que el paciente pueda ser trasladado a otra unidad sanitaria de mayor complejidad, con vistas a continuar el tratamiento. Independientemente del tipo de asistencia prestada (SVA o SVB), cabe destacar la responsabilidad de los equipos a la hora de prestar asistencia a las personas afectadas por problemas de salud, estabilizarlas y derivarlas con seguridad y calidad a los centros sanitarios^(6)^.

Los datos generados durante cada servicio (información sobre el paciente, el incidente y los procedimientos realizados) se registran en las Fichas Sistematizadas de Servicios (FASis), que proporcionan respaldo jurídico al equipo y al paciente, así como visibilidad al servicio prestado, apoyando los análisis epidemiológicos esenciales para mejorar la APH local y su continuidad^(7)^.

En 2023, el SAMU estaba presente en el 67,3% de los municipios brasileños, atendiendo al 85% de la población^(8)^. Su financiación ha sido responsabilidad tripartita del gobierno Federal, con el 50% de los costes, los estados y los municipios, que sufragan los gastos según el acuerdo estipulado en cada Comisión Interinstitucional Bipartita^(3)^.

Para la construcción de Centros de Regulación Médica de Urgencia (CRMU) y la ampliación de los ya existentes, los fondos se asignan de acuerdo con los siguientes parámetros: R$216.000,00, cuando la población municipal sea de hasta 350.000 habitantes; R$350.000,00, cuando la población municipal esté entre 350.001 y 3.000.000 de habitantes; R$440.000,00, cuando la población municipal sea de más de 3.000.000 de habitantes^(9)^. Los parámetros de los costes mensuales son los siguientes R$54.600,00 mensuales, cuando calificado; y R$68.386,50 mensuales, cuando calificado y con hasta 350.000 habitantes^(10)^.

Los importes para la adquisición de tecnología y equipos informáticos para las CRMUs cubren hasta R$96.847,21 cuando la población municipal es de hasta 350.000 habitantes; hasta R$288.817,15 cuando la población municipal está entre 350.001 y 10.000.000 habitantes; y R$302.902,15 cuando la población municipal es superior a 10.000.001 habitantes^(9)^.

Para el mantenimiento de los equipos efectivamente constituidos, se han establecido los siguientes importes: 17.062,50 reales mensuales por unidad SVB autorizada; 28.494,70 reales mensuales por unidad SVB autorizada y cualificada; 50.050,00 reales mensuales por unidad SVA autorizada; 62.687,30 reales mensuales por unidad SVA cualificada; 54.600 reales mensuales (población de hasta 350.000 habitantes) por unidad CRMU cualificada; y 68.386,50 reales mensuales (población de hasta 350.000 habitantes) por unidad CRMU cualificada.687,30 al mes por unidad SVA habilitada; 54.600 reales al mes (población de hasta 350.000 habitantes) por unidad CRMU habilitada; y 68.386,50 reales al mes (población de hasta 350.000 habitantes) por unidad CRMU habilitada^(9)^.

La financiación mensual asignada al SAMU esta directamente vinculada a los registros mensuales de asistencias, y podrá suspenderse cuando no se cumplan los requisitos de calificación, el registro de asistencias sea inferior al establecido por la ordenanza específica de la Secretarla de Asistencia Sanitaria, si no hay registros de asistencias durante tres meses consecutivos o si se detectan irregularidades en el servicio^(9)^.

La gestión municipal es responsable de proporcionar los recursos materiales, humanos y financieros necesarios para que el SAMU sea viable, de modo que no se ponga en peligro la calidad de la asistencia prestada^(3)^. Cabe destacar que los municipios se han animado a implantar el SAMU debido al incentivo financiero para su puesta en marcha, además de la distribución de vehículos equipados^(9)^. Según un estudio realizado en 2022, el SAMU registró 133,4 millones de procedimientos entre 2015 y 2019. La población cubierta por aumentó un 9,7%; el número de llamadas a través de 192 aumentó un 26,5%; y el número total de procedimientos realizados por SVB y SVA aumentó un 28,5%^(11)^.

La política nacional de ampliación de los servicios de urgencia y emergencia requiere una financiación compatible con las exigencias del SUS, en referencia a la suficiencia y distribución adecuada de los recursos^(12)^. Se sabe que una buena gestión es una base estratégica para regular y organizar la asistencia prestada por el SAMU. Sin embargo, existen algunos problemas en el sistema de salud que pueden repercutir en la calidad de la atención, como la inversión inadecuada, la precariedad de los recursos, la falta de infraestructura, la falta de recursos humanos y la falta de mantenimiento de los vehículos, por lo que es necesario que las autoridades de gestión se comprometan a realizar las inversiones necesarias^(13)^.

Un estudio realizado en 2022, en la región del Gran ABC de São Paulo, mostró que los gestores destacaban el aumento de la demanda del SAMU, pero sin aumento de la financiación. También destacaron la falta de equipos y suministros necesarios para el servicio, subrayando que la falta de recursos poda comprometer la obtención de resultados favorables^(14)^.

Teniendo en cuenta que los recursos financieros son limitados y que la demanda de atención es creciente, un paso importante en la gestión de costes es calcular los costes asociados a la APH. El objetivo era, por tanto, identificar los costes directos medios (CDM) de personal, materiales, medicamentos y soluciones necesarios para la APH del SAMU en un municipio de São Paulo.

## MÉTODO

### Tipo o Diseño del Estudio

Se trata de un estudio de microcostos *ascendente* por absorción retrospectiva en forma de estudio de caso único*.*

### Ubicación

La investigación se llevó a cabo en la Base Regional del SAMU de un municipio brasileño del estado de São Paulo. En 2022, este SAMU recibió 85.709 llamadas (75.429 clínicas, 5.964 traumatológicas, 1.202 obstétricas, 1.137 pediátricas y 1.977 psiquiátricas), que dieron lugar a 28.267 asistencias *in situ*, de las cuales 26.177 fueron SVB y 2.090 SVA.

### Muestra y Criterios de Selección

A partir de la población de FASis (N = 28.267) para el año 2022, un estadístico calculó el tamaño mínimo de la muestra que debía analizarse, utilizando la metodología de dimensionamiento de muestras para estimar una media. Como no se disponía de una estimación de la desviación estándar (DE) del coste, se asumió un margen de error ε = 0,1σ, es decir, un máximo del 10% de la DE. Esto dio lugar a un cálculo de la muestra de 380 FASis, 188 relacionados con la atención prestada por los equipos SVA y 192 por los equipos SVB.

Se seleccionaron los FASis que estaban correctamente cumplimentados con las variables de identificación de la llamada (municipio, fecha, hora, vehículo llamado) y del paciente (sexo y edad), el lugar del incidente, tiempo empleado y los suministros (materiales, medicamentos y soluciones) consumidos. Se excluyeron los FASis cumplimentados de forma incompleta.

### Recogida de Datos

Todos los datos, incluidos los relativos al tiempo de atención de los profesionales sanitarios, se recogieron entre mayo y agosto de 2023 accediendo electrónicamente a la muestra de FASis cumplimentadas por los equipos SVA y SVB en la sala de Coordinación Administrativa de la Gerencia Municipal de Salud.

El estudio consistió en un estudio de microcostos ascendente por absorción, en el que se consultaron los recursos reales consumidos por un paciente o servicio sanitario, determinando el coste real de la prestación de asistencia sanitaria por parte de un proveedor. Se eligió el método de microcosteo *ascendente* porque permite definir en detalle todas las variables componentes del costo, a partir de los datos de tratamiento de pacientes individuales, mediante la revisión de historias clínicas o registros clínicos específicos para el estudio^(15)^.

El cálculo de costes por absorción corresponde a la imputación de los costes de producción a los bienes producidos. De este modo, todos los costes relacionados con la producción se reparten entre los servicios o productos fabricados; esto significa que los costes de fabricación se asignan al coste del producto^(16)^.

Así, se calcularon los costes directos, definidos como aquellos que pueden ser identificados y cuantificados, que se refieren a un valor monetario relativo al consumo de mano de obra directa (MOD), materiales, medicamentos y soluciones^(16)^. La MOD se refiere a los profesionales que trabajan en un determinado producto/servicio, siempre que sea posible medir el tiempo consumido en su realización. Se compone de sueldo, cargas, vacaciones y decimotercer sueldo^(6)^.

La MOD de los profesionales de salud se calculó a partir de la información salarial proporcionada por la Dirección Municipal de Recursos Humanos (DRH) responsable del SAMU estudiado. Para los costos, los valores obtenidos en reales brasileños (R$) fueron convertidos en dólares estadounidenses (US$) a una tasa promedio de US$4,75/R$1,00, con base en las tasas de cambio de enero a diciembre de 2022 proporcionadas por el Banco Central de Brasil.

Así, la masa salarial media ponderada de los médicos fue de 252,63 US$/12 horas, 21,05 US$/hora y 0,35 US$/minuto; la de los enfermeros fue de 105,26 US$/12 horas, 8,77 US$/hora y 0,15 US$/minuto; y la de los técnicos de enfermería fue de 52,63 US$/12 horas, 4,38 US$/hora y 0,07 US$/minuto. La información sobre los costes de las últimas compras de insumos materiales, medicamentos y soluciones fue facilitada por el Departamento de Compras/Almacenamiento.

Cabe señalar que, durante el período en que se realizó el estudio, la conducción de los vehículos SVA y SVB era una actividad delegada a los bomberos militares remunerados por el Estado, de cuya información salarial no disponía el citado DRH. No fue posible cubrir el coste del consumo de combustible de los vehículos, ya que todos los vehículos del municipio se abastecían en el mismo lugar y el pago se realizaba en un único pago global, lo que hacía imposible distinguir los vehículos de los demás.

Para obtener el CDM de las atenciones registradas por los equipos SVA y SVB, se utilizó la ecuación^(17)^ $\bar{C\left(P_{\text{τ}}\right)}=\sum_{k=1}^{n}\left(\bar{q_{k}.}\;\bar{Pu_{k}}\right)+\sum_{k=1}^{n}\left(\bar{qs_{k}.}\;\bar{Psu_{k}}\right)+\sum_{c=1}^{n}\left(\bar{t_{c}.}\;\bar{su_{c}}\right)$ , en la que se incluyeron las siguientes variables: $\bar{qm_{k}}$ (cantidad media de materiales); $\bar{Pmu_{k}}$ (precio unitario de cada material); $\bar{qs_{k}}$ (cantidad media de soluciones/medicamentos); $\bar{Psu_{k}}$ (precio unitario de cada solución/medicamento); $\bar{t_{c}}$ (tiempo medio empleado por cada profesional); $\bar{Su_{c}}$ (coste salarial unitario de cada mano de obra)^(17)^. En resumen, el tiempo empleado por el profesional actuante se multiplicó, según la categoría, por el coste del MOD respectivo, más el coste de los materiales, medicamentos y soluciones^(1(7) ()))^consumidos en cada servicio de SVA y SVB.

### Análisis y Tratamiento de Datos

Los datos se organizaron en una hoja de cálculo electrónica con doble mecanografía independiente, que luego se procesó mediante estadísticas descriptivas e inferenciales.

### Aspectos Éticos

El Comité de Ética de la Investigación de la institución proponente aprobó el estudio tras obtener el consentimiento del Departamento Municipal de Salud responsable del SAMU estudiado (Dictamen n^º^ 6.031.451, de 28 de abril de 2023).

## RESULTADOS

A partir de la muestra de 192 FASis referentes a los cuidados prestados por los equipos SVB, el tiempo medio empleado por el técnico de enfermería fue de 15,85 minutos. La Tabla 1 muestra que el CDM total correspondió a US$3,21/servicio (DE = 2,61), con la mayor representación del CDM para materiales, medicamentos y soluciones (US$2,10-DP = 2,26).

**Tabla 1:** Distribución de los costos laborales directos en dólares (US$*) de los técnicos de enfermería que trabajan en 192 visitas de equipos de Soporte Vital Básico según media, desvío estándar, mínimo, máximo, 1º cuartil, mediana y 3º cuartil – São Paulo, SP, Brasil, 2023

Variables	CDM (US$*)	Desviación típica	Porcentaje de la composición del CDM total (%)	Mínimo (US$*)	Máximo (US$*)	1er cuartil (US$)	Mediana (US$)	3er cuartil (US$)
**Total CDM con técnico de enfermería MOD**	**1,11**	**0,75**	**34,55%**	0,15	4,82	0,58	0,95	1,41
**CDM total con materiales, medicamentos y soluciones**	**2,10**	**2,26**	**65,45%**	0,00	18,18	0,41	1,68	3,05
**CDM total por servicio SVB**	**3,21**	**2,61**	**100,00%**	0,15	19,94	0,34	2,39	4,40

Nota: *Tipo de conversión medio: R$ 4,75/US$ 1,00. Banco Central, basado en el tipo de cambio de enero a diciembre de 2022; MOD: mano de obra directa; SVB: soporte vital básico; CDM: coste directo medio.

De acuerdo con los registros de materiales, medicamentos y soluciones contenidos en los 192 FASis analizados, el costo total de la atención en SVB fue de US$396,67 (100,00%), con un CDM de US$2,10 por atención. La Tabla 2 muestra que de los 39 ítems, los que más contribuyeron para ese costo total fueron collarín cervical P/33 atenciones (costo unitario: US$3,00; 24,94%), solución fisiológica 0,9% 250 ml/40 atenciones (costo unitario: US$0,93; 9,19%), férula M/14 atenciones (costo unitario: US$2,57; 9,07%) y collarín cervical M/10 atenciones (costo unitario: US$3,19; 8,04%).

**Tabla 2:** Distribución de los costos en dólares (US$*) de materiales, medicamentos y soluciones consumidos en 192 consultas de Soporte Vital Básico según costo total, porcentaje del costo total, número de consultas, costo directo promedio por consulta y valor unitario *–* São Paulo, SP, Brasil, 2023.

Materiales, medicamentos y soluciones (insumos)	Coste total (US$)	Porcentaje de la composición del coste total (%)	Número de asistencias	CDM de insumos por servicio (US$)	Coste unitario del insumo (US$)
Collarín cervical P	98,93	24,94	33	3,00	3,00
Solución fisiológica 0,9% 250 ml	36,45	9,19	40	0,93	0,93
Férula con cable M	35,99	9,07	14	2,57	2,57
Collarín cervical M	31,89	8,04	10	3,19	3,19
Tubo de macrogoteo	22,05	5,56	38	0,61	0,61
Máscara de alta concentración	20,77	5,24	8	2,60	2,60
Férula con cable – G	18,91	4,77	5	3,78	3,78
Solución fisiológica 0,9% 500 ml	16,45	4,15	17	0,97	1,10
Férula con cable – P	15,24	3,84	6	2,54	2,18
Venda de 12 cm	13,60	3,43	53	0,26	0,16
Tira de glucosa en sangre capilar	13,41	3,38	93	0,15	0,15
Gasa de algodón	11,76	2,96	12	0,98	0,73
Férula con cables – GG	8,11	2,05	1	8,11	4,06
Catéter sin aguja 20	7,12	1,80	18	0,42	0,42
Catéter sin aguja 22	6,25	1,58	15	0,45	0,45
Sonda nasal tipo gafa	5,46	1,38	32	0,17	0,17
Guante de procedimiento – P (látex/polvo)	4,71	1,19	89	0,05	0,03
Glucosa 50% ampolla 10 ml	4,28	1,08	12	0,36	0,12
Gasa estéril (paquete)	3,82	0,96	27	0,14	0,14
Guante de procedimiento – M (látex/polvo)	3,69	0,93	74	0,03	0,03
Lanceta para la glucemia capilar	3,07	0,77	93	0,03	0,03
Venda triangular - M	2,88	0,73	4	0,72	0,72
Catéter de aguja 21	2,15	0,54	5	0,43	0,43
Catéter de aguja 23	2,09	0,53	5	0,42	0,42
Catéter sin aguja 18	1,89	0,48	4	0,47	0,47
Guante de procedimiento – G (látex/polvo)	1,30	0,33	28	0,05	0,02
Jeringa de 20 ml	1,15	0,29	14	0,07	0,07
Catéter sin aguja 14	0,95	0,24	1	0,95	0,95
Catéter sin aguja 24	0,49	0,12	1	0,49	0,49
Cánula Guedel 4	0,44	0,11	1	0,44	0,44
Dipirona sódica 500 mg ampolla	0,23	0,06	1	0,23	0,23
Aguja 40x12	0,20	0,05	15	0,01	0,01
Mascarilla N95	0,20	0,05	1	0,20	0,20
Electrodos de control	0,20	0,05	1	0,20	0,04
Ampolla de butilbromuro de escopolamina	0,20	0,05	1	0,20	0,20
Jeringa de 10 ml	0,12	0,03	2	0,06	0,04
Captopril 25 mg comprimido	0,12	0,03	4	0,02	0,02
Ampolla de agua destilada 10 ml	0,11	0,03	1	0,11	0,05
Monos desechables	0,01	0,00	1	0,01	0,01

Nota: *Tipo de conversión medio: R$ 4,75/US$ 1,00. Banco Central, basado en el tipo de cambio de enero a diciembre de 2022; MOD: mano de obra directa; CDM: coste directo medio.

Considerando la muestra de 188 FASis relacionadas a la atención prestada por los equipos de los SVA, la Tabla 3 muestra que el CDM total fue de US$19,70/servicio (DE = 36,34), con la mayor representación del CDM total con MOD de los profesionales de salud (US$10,99–DP = 5,81), cuyo tiempo medio de atención conjunta fue de 22 minutos.

**Tabla 3:** Distribución de los costos en dólares (US$*) de la mano de obra directa de los profesionales de salud que atienden 188 llamadas de Soporte Vital Avanzado y de los insumos consumidos según media, desvío estándar, mínimo, máximo, 1º cuartil, mediana y 3º cuartil – São Paulo, SP, Brasil, 2023.

Variables	CDM (US$*)	Desviación típica	Porcentaje de la composición del CDM total (%)	Mínimo (US$*)	Máximo (US$*)	1er cuartil (US$)	Mediana (US$)	3er cuartil (US$)
CDM con MOD de enfermera	3,23	2,12	16,40	0,58	15,79	1,90	3,80	3,80
CDM con MOD de médico	7,75	5,10	39,36	1,40	37,89	4,56	6,67	9,12
**Total CDM con MOD**	**10,99**	**5,81**	**55,76**	1,99	53,68	6,46	9,44	12,92
**CDM total con materiales, medicamentos y soluciones**	**8,72**	**32,06**	**44,24**	0,27	212,46	2,24	3,02	5,12
**CDM total de asistencia por SVA**	**19,70**	**36,64**	**100,00**	**2,79**	**256,51**	**2,24**	**13,27**	**18,05**

Nota: *Tipo de conversión medio: R$ 4,75/US$ 1,00. Banco Central, basado en el tipo de cambio de enero a diciembre de 2022; MOD: mano de obra directa; SVA: Soporte Vital Avanzado; CDM: coste directo medio.

De acuerdo con los registros de los materiales, medicamentos y soluciones contenidos en los 188 FASis, el costo total fue de US$1.647,49 (100,00%), con un CDM de US$$ 8,72 por servicio. Según la Tabla 4, de los 73 ítems consumidos, los que más contribuyeron para ese costo total fueron el kit de punción intraósea/cinco visitas (costo unitario: US$193,68; 58,78%), solución fisiológica 0,9%–500 ml/75 visitas (costo unitario: US$1,10; 5,33%), collarín cervical P/27 visitas (coste unitario: US$3,00; 4,91%), equipo de macrogoteo/130 visitas (coste unitario: US$0,61; 4,91%) y mascarilla de alta concentración/21 visitas (coste unitario: US$2,60; 3,31%).

**Tabla 4:** Distribución de los costos en dólares (US$) de materiales, medicamentos y soluciones consumidos en 188 visitas de Soporte Vital Avanzado según costo total, porcentaje del costo total, número de visitas, costo directo promedio por visita y valor unitario – São Paulo, SP, Brasil, 2023.

Materiales, medicamentos y soluciones (insumos)	Coste total (US$)	Porcentaje de la composición del coste total (%)	Número de asistencias	CDM de insumos por servicio (US$)	Coste unitario del insumo (US$)
Kit de punción intraósea	968,42	58,78	5	193,68	193,68
Solución fisiológica 0,9% 500 ml	87,75	5,33	75	1,17	1,10
Collarín cervical P	80,94	4,91	27	3,00	3,00
Tubo de macrogoteo	80,87	4,91	130	0,61	0,61
Máscara de alta concentración	54,51	3,31	21	2,60	2,60
Solución de Ringer lactato 500 ml	38,50	2,34	22	1,75	1,60
Solución fisiológica 0,9% 250 ml	35,52	2,16	35	1,01	0,93
Férula con cables – M	23,13	1,40	8	2,89	2,57
Electrodos de control	20,60	1,25	103	0,20	0,04
Catéter sin aguja 20	20,11	1,22	48	0,42	0,42
Gasa de algodón	19,10	1,16	16	1,19	0,73
Férula con cable – G	18,91	1,15%	4	4,73	3,78
Catéter sin aguja 18	15,09	0,92	32	0,47	0,47
Catéter sin aguja 22	13,39	0,81	30	0,45	0,45
Solución de glucosa al 5% 250 ml	10,93	0,66	9	1,21	1,09
Venda de 12 cm	9,12	0,55	32	0,29	0,16
Catéter sin aguja 16	8,70	0,53	13	0,67	0,67
Tira de glucosa en sangre capilar	8,55	0,52	58	0,15	0,15
Guante de procedimiento M (látex/polvo)	8,24	0,50	108	0,08	0,03
Epinefrina 1 mg ampolla	7,52	0,46	9	0,84	0,21
Guante de procedimiento – P (látex/polvo)	6,95	0,42	89	0,08	0,03
Férula con cable P	6,53	0,40	3	2,18	2,18
Tubo orotraqueal 7,5	6,46	0,39	9	0,72	0,72
Glucosa 50% ampolla 10 ml	6,25	0,38	14	0,45	0,12
Cloruro de suxametonio 100 mg ampolla	5,91	0,36	2	2,95	2,95
Midazolam 15 mg ampolla	4,21	0,26	7	0,60	0,60
Midazolam 5 mg ampolla	4,04	0,25	5	0,81	0,40
Guante de procedimiento G (látex/polvo)	3,98	0,24	64	0,06	0,02
Dipirona sódica 500 mg ampolla	3,83	0,23	17	0,23	0,23
Etomidato ampolla 10 ml	3,83	0,23	2	1,91	1,91
Sonda nasal tipo gafa	3,75	0,23	22	0,17	0,17
Catéter sin aguja 24	3,45	0,21	7	0,49	0,49
Ondansetrón ampolla 2 ml	3,27	0,20	8	0,41	0,41
Tramadol clorhidrato ampolla	3,23	0,20	7	0,46	0,46
Collarín cervical M	3,19	0,19	1	3,19	3,19
Haloperidol 5 mg ampolla	3,09	0,19	8	0,39	0,34
Ketoprofeno ampolla vial	2,77	0,17	4	0,69	0,69
Gasa estéril (paquete)	2,68	0,16	14	0,10	0,10
Catéter de aguja 23	2,51	0,15	6	0,42	0,42
Flumazenil 0,5 mg ampolla	2,37	0,14	2	1,19	1,19
Omeprazol 40 mg ampolla	2,27	0,14	2	1,14	1,14
Venda triangular – M	2,16	0,13	3	0,72	0,72
Catéter de aguja 21	2,15	0,13	5	0,43	0,43
Ampolla de diazepan	2,15	0,13	10	0,21	0,21
Tubo orotraqueal 8,0	2,15	0,12	3	0,67	0,67
Lanceta para la glucemia capilar	1,95	0,12	58	0,03	0,03
Catéter sin aguja 14	1,89	0,12	2	0,95	0,95
Ampolla de morfina	1,83	0,11	2	0,92	0,92
Ampolla de 50 mcg de citrato de fentanilo	1,81	0,11	4	0,45	0,45
Clopidogrel 75 mg comprimido	1,77	0,11	6	0,29	0,07
Ampolla de fenitoína	1,52	0,09	3	1,03	0,60
Amiodarona 50 mg ampolla	1,41	0,09	2	0,71	0,47
Jeringa de 5 ml	1,37	0,08	33	0,04	0,03
Cánula Guedel 4	1,33	0,08	3	0,44	0,44
Jeringa de 10 ml	1,28	0,08	30	0,04	0,04
Jeringa de 20 ml	1,28	0,08	15	0,09	0,07
Ampolla de clorhidrato de prometazina	1,14	0,07	3	0,38	0,38
Aguja 40 × 12	0,92	0,06	69	0,01	0,01
Ampolla de agua destilada 10 ml	0,89	0,05	13	0,07	0,05
Ácido tranexámico ampolla	0,86	0,05	1	0,86	0,86
Conector multifilar	0,77	0,05	3	0,26	0,26
Manta térmica – G	0,49	0,03	1	0,49	0,49
Ampolla de furosemida	0,47	0,03	1	0,47	0,24
Ampolla de butilbromuro de escopolamina	0,40	0,02	2	0,20	0,20
Aguja 25 × 7	0,34	0,02	13	0,03	0,02
Mascarilla N95	0,20	0,01	1	0,20	0,20
Captopril 25 mg comp.	0,19	0,01	6	0,03	0,02
Ácido acetilsalicílico 100 mg comprimido	0,18	0,01	8	0,02	0,01
Tubo de aspiración traqueal 10	0,12	0,01	1	0,12	0,12
Diazepan 10 mg comprimido	0,07	0,00	4	0,02	0,02
Isosorbida 5 mg comprimido	0,04	0,00	3	0,01	0,01
Monos desechables	0,03	0,00	2	0,01	0,01
Comprimido de hidroclorotiazida	0,01	0,00	1	0,01	0,01

Nota: *Tipo de conversión medio: R$ 4,75/US$ 1,00. Banco Central, basado en el tipo de cambio de enero a diciembre de 2022; MOD: mano de obra directa; SAV: Soporte Vital Avanzado; CDM: coste directo medio.

Los CDM totales para la muestra de 380 FASis ascendieron a 4.319,92 US$, de los cuales 616,32 US$ correspondían a 192 casos de SVB y 3.703,60 US$ a 188 casos de SVA. Extrapolando los CDM a la población de 28.267 FASis, el total de CDM en 2022 sería de 125.201,17 US$ (100,00%), de los cuales 84.028,17 US$ (67,11%) se deberían a 26.177 casos de SVB y 41.173,00 US$ (32,89%) a 2.090 casos de SVA.

## DISCUSIÓN

En la atención prestada por los equipos SVB, el CDM de materiales, medicamentos y soluciones fue superior al CDM de la MOD de los técnicos de enfermería, aportando el 65,45% del CDM total por atención. Los materiales más utilizados fueron las lancetas y las tiras de glucemia capilar, que se utilizaron en 93 de las 192 asistencias. Estudios recientes, que abarcan diferentes temas, también han indicado que predominan los costes relacionados con los recursos materiales^(18-20)^.

Un estudio de caso único, cuantitativo, exploratorio-descriptivo, que calculó el CDM para la inserción de un catéter venoso central de larga duración en pacientes en hemodiálisis en un hospital público del estado de São Paulo, obtuvo un CDM de 107,01 US$ (DE = 0,23) para materiales, 22,10 US$ (DE = 3,63) para MOD de profesionales, 4,65 US$ (DE = 0,00) para medicamentos y 0,80 US$ (DE = 0,15) para soluciones^(18)^.

Otro estudio cuantitativo, exploratorio-descriptivo, de caso único, en el que se calculó el CDM del tratamiento del cáncer de próstata mediante *High Intensity Focused Ultrasound* en un centro quirúrgico, mostró que los costes de materiales (851,58 USD – DE = 2,17) tenían un mayor impacto en el CDM total en comparación con el CDM de fármacos/soluciones (72,13 USD – DE = 25,84)^(19)^.

Un estudio cuantitativo, cuyo objetivo era estimar el CDM para la inserción de un catéter central de inserción periférica en pacientes adultos en una unidad de cuidados intensivos cardiopulmonares de un hospital universitario público, midió el CDM en 259,81 USD (DE ± 36,94), en relación con los materiales, y 26,22 USD (DE ± 9,01), en relación con la MOD de las enfermeras actuantes^(20)^.

En el caso de los equipos de SVA, los CDM de la MOD de médicos y enfermeros se solapaban con los CDM de materiales, medicamentos y soluciones. Cabe señalar que, a pesar de las frecuentes búsquedas en la literatura, no se encontraron estudios que hubieran analizado los costes asociados a la MOD de los profesionales sanitarios que trabajan en SAMU. En cuanto a los estudios de microcostos *ascendentes*, además de escasos, pocos muestran la prevalencia de los costes de la MOD de recursos humanos en relación con los costes de materiales, medicamentos y soluciones^(21,22)^.

Se trata de un estudio cuantitativo, transversal, analítico, con análisis de microcosteo por absorción, realizado en el Banco de Leche Humana de un hospital universitario del sur de Brasil, sobre la aplicación de terapia láser local y transcutánea por *intravascular laser irradiation of blood* en el tratamiento de traumatismos mamarios, mostraron que, en los tres grupos estudiados (grupo control, grupo láser local y grupo *intravascular laser irradiation of blood*), el coste de la MOD de enfermería fue el más representativo del coste total del procedimiento, oscilando entre el 76,00% y el 94,00%^(21)^.

Un estudio cuantitativo, exploratorio-descriptivo, de caso único, realizado en un centro de diálisis de un hospital público docente y de investigación, midió el CDM de los procedimientos realizados para el manejo de las complicaciones del acceso vascular para hemodiálisis convencional. Se observó que, en la mayoría de los procedimientos, el CDM con MOD de profesionales de enfermería predominaba en la composición del CDM total. En la administración de antibióticos de fácil dilución, la administración de antibióticos de difícil dilución, la administración de antibióticos sin diluir, el apósito con antibióticos tópicos en el lugar de inserción del catéter venoso central y la punción de una fístula arteriovenosa, el CDM de los profesionales fue superior al CDM de los materiales, medicamentos y soluciones^(22)^.

En los cuidados de SVA, el *kit de* punción intraósea fue el material que más contribuyó al coste directo total. Aunque este artículo sólo se utilizó en cinco de las 188 visitas, tuvo un coste unitario elevado (US$ 193,68), correspondiente al 58,78% del coste total de materiales, medicamentos y soluciones. A pesar del elevado coste unitario, este *kit*, disponible únicamente en el vehículo SVA, se considera un artículo relevante, ya que permite una intervención rápida en una situación de emergencia cuando el acceso venoso periférico es imposible, favoreciendo el establecimiento de una vía de administración de soluciones y medicamentos^(23)^.

A pesar de su bajo valor unitario (US$ 0,61), las macrogotas fueron el insumo más utilizado en 130 de los 188 procedimientos de SVA y aportaron el 4,91% del coste total de los insumos. Hubo una mayor variedad en el consumo de materiales en las visitas de SVA, ya que implican procedimientos más invasivos y complejos. Así, en una misma visita, el equipo realizó desde inmovilizaciones, controles de glucemia capilar y curas hasta punciones intraóseas, intubaciones orotraqueales y administración de medicación. A pesar de esta variedad, cabe reiterar que de los 19,70 dólares (DE = 36,64) de CDM por servicio de SVA, el coste de los materiales representó el 44,24%, predominando el CDM con MOD (55,76%).

Los precios de compra de los materiales, medicamentos y soluciones consumidos en los servicios SVB y SVA fueron inferiores a los practicados en el mercado, debido al proceso de adquisición mediante licitación, en el que la Administración Pública compra materiales y/o suministros, y contrata servicios. Sin embargo, si se hubiera aplicado ampliamente la Ley de Licitaciones y Contratos nº 14.133^(24)^, los precios habrían sido diferentes. Esta nueva ley se caracteriza por una serie de innovaciones, como la exclusión de las modalidades de carta de invitación y cotización de precios, y la inclusión de una nueva modalidad: el diálogo competitivo. Posibilita el uso de sistemas electrónicos de licitación, lo que facilita la participación de empresas de todos los tamaños y de distintas regiones del país. También hace uso del arbitraje en caso de controversias e incluye criterios de desempate además del precio, lo que permite una mayor competitividad entre las empresas participantes^(24)^.

Cabe destacar que la identificación de los costes de los recursos materiales proporciona a las enfermeras conocimientos específicos que ayudan a reforzar los argumentos sobre la rentabilidad de su adquisición, con vistas a prestar servicios sanitarios seguros y de calidad y llevar a cabo con éxito los procesos de licitación^(25)^.

Dadas las repercusiones financieras asociadas al consumo de recursos materiales, especialmente en los servicios públicos con presupuestos restringidos, es imprescindible mejorar su gestión, que se ha visto incrementada con la participación cualificada de las enfermeras, que han contribuido a hacer posible el suministro de materiales en cantidad y calidad suficientes para prestar unos cuidados seguros y de calidad^(25,26)^.

Esto es corroborado por el hecho de que la gestión de costes puede ayudar a evaluar un determinado tipo de prestación de servicios sanitarios, así como su repercusión en los resultados que deben alcanzarse. Por lo tanto, se ha convertido en una herramienta esencial para las organizaciones sanitarias, dado que a medida que aumenta la demanda de asistencia, también lo hacen los costes asociados al tratamiento. Por ello, es necesario y pertinente que los profesionales sanitarios hagan suyo el conocimiento de los costes sanitarios y, a partir de él, busquen posibilidades que garanticen su aplicabilidad para maximizar resultados satisfactorios en las condiciones de salud de la población, dados los limitados y restringidos recursos disponibles^(27)^.

En la dimensión macroeconómica, la gobernanza de la financiación de una red asistencial se realiza a través de planes de actuación regional, detallando los recursos financieros, la responsabilidad de cada entidad en la sostenibilidad de los planes y los recursos que serán asignados por los prestadores de servicios de salud. Se hace operativa a través de instrumentos y mecanismos de regulación de la atención, como la programación general, la contratación de establecimientos públicos y/o prestadores privados y la regulación del acceso^(28)^.

Desde esta perspectiva, un estudio de caso que evaluó el sistema de gobernanza para la financiación del componente prehospitalario móvil de la Red de Atención de Urgencia y Emergencia en una región de salud del estado de Paraná, infirió lo incipiente que es la gobernanza del SAMU, especialmente en lo que se refiere a la financiación, con poco detalle sobre los recursos financieros, incluyendo cuánto recibe el municipio en incentivos de otras entidades, cuánto invierte en recursos propios para mantener el servicio, así como la falta de espacios efectivos de gobernanza en esta red. Se concluye que la deficiencia en la gobernanza de la financiación del SAMU aumenta su dificultad para organizar y articular la integración de los componentes de la Red de Atención de Urgencias y Emergencias^(29)^.

A pesar de los avances del gobierno en la creación de un bloque específico de recursos vinculados a las acciones de emergencia, todavía hay desafíos considerables para la plena gestión del SAMU en todos los niveles de gobierno, con la necesidad de regulación e integración efectiva entre los otros niveles de atención. Un estudio que describió y analizó la evolución del gasto público federal en SAMU, comparando municipios, regiones y macrorregiones sanitarias en el estado de Bahía entre 2009 y 2012, destacó en su momento la necesidad de aumentar la financiación del SAMU y de distribuir mejor los recursos, reparando las desigualdades existentes entre las macrorregiones de salud y las regiones, así como la necesidad de una mayor inversión en la estructura y organización de la red de atención del SUS, con el fin de absorber con resolutividad las demandas derivadas del rescate, garantizando una atención integral^(12)^.

### Implicaciones Prácticas

Este estudio representa un avance en el conocimiento al abordar los aspectos financieros asociados a la atención prestada por los equipos de SVB y SVA de un SAMU. Ante la falta de estudios de esta naturaleza, los resultados obtenidos pueden contribuir a fundamentar las decisiones asistenciales y de gestión en cuanto a la asignación racional de los profesionales sanitarios, materiales, medicamentos y soluciones necesarias, aumentando su potencial y evitando la aparición de despilfarros y riesgos para la calidad de la atención prestada a los usuarios.

### Limitaciones del Estudio

Las limitaciones de este estudio incluyen el hecho de que no se recogieron datos de observaciones no participantes y la imposibilidad de costear la MOD de los conductores de los vehículos SVA y SVB, así como el consumo de combustible que requieren estos vehículos.

## CONCLUSIÓN

En el caso de la atención prestada por los equipos SVB, los CDM relativos a materiales, medicamentos y soluciones (US$2,10) fueron más representativos en la composición del CDM total (US$3,21), a diferencia de los servicios prestados por los equipos SVA, en los que predominaron, en el CDM total (US$ 19,70), los CDM relativos a la MOD de médicos y enfermeros (US$10,98 – DP = 5,81).

Esto dio como resultado un CDM total para la muestra de 380 FASis de 4.319,92 USD (100,00%), de los cuales el 85,73% correspondían a las 188 asistencias de SVA y el 14,27% a asistencias de SVB. Extrapolando los CDM a la población de 28.267 FASis, el total de CDM para la atención prestada por los equipos SAMU en 2022 sería de 125.201,17 US$ (100,00%), de los cuales el 67,11% estarían relacionados con 26.177 asistencias SVB y el 32,89% con 2.090 asistencias SVA.

La identificación de los costes de la APH proporcionada por los equipos de SVB y SVA tiene el potencial de dar aportes para las decisiones estratégicas de atención y gestión dirigidas a la sostenibilidad financiera y económica del SAMU.

## Data Availability

Se trata de un estudio de caso único, cuyos datos que respaldan los resultados no están disponibles públicamente debido a la presencia de información sensible y a la necesidad de preservar la confidencialidad de los participantes. Toda la información relevante para comprender los hallazgos se describe en el cuerpo del artículo.
